# Exposure to Heavy Metals Arsenic, Cadmium and Lead Exacerbates Carcinogenic and Noncarcinogenic Health Risks Among Diabetic and Hypertensive Patients

**DOI:** 10.1155/jt/8564316

**Published:** 2026-06-30

**Authors:** Fombat Zenabou Mbebwoh, Muankang Junior Tegha Kum, Choumessi Tchewonpi Aphrodite, Kada Sanda Antoine, Fusi Christian Suh, Manfo Tsague Faustin Pascal, Nantia Akono Edouard

**Affiliations:** ^1^ Department of Biochemistry, Faculty of Science, University of Bamenda, PO Box 39, Bambili, Cameroon, unibda.net; ^2^ Department of Zoology, Faculty of Science, University of Bamenda, PO Box 39, Bambili, Cameroon, unibda.net; ^3^ Department of Biochemistry and Molecular Biology, Faculty of Science, University of Buea, PO Box 63, Buea, Cameroon, ubuea.cm

**Keywords:** diabetes, heavy metals, hypertension, oxidative stress, predictive analysis, risk assessment

## Abstract

Exposure to toxic metals contributes to the growing burden of noncommunicable diseases (NCDs), yet evidence from sub‐Saharan Africa remains limited. This hospital‐based cross‐sectional study assessed heavy metal (arsenic [As], cadmium [Cd], lead [Pb]) exposure levels and their association with health risks in adult diabetic and hypertensive patients in Cameroon. It involved 178 participants (39 diabetics, 78 hypertensives, 61 comorbid), and the urinary metals were quantified using atomic absorption spectrophotometry and normalized to creatinine. Serum oxidative stress biomarkers including glutathione (GSH), thiobarbituric acid reactive substances (TBARS) and paraoxonase 1 (PON1) were measured. Health risk indices including estimated daily intake (EDI), chronic daily intake (CDI), hazard quotient (HQ), hazard index (HI) and cancer risk (CR), were computed. Proportion contribution trend (PCT), receiver operating characteristic (ROC) and ordinary least squares (OLS) regression were applied for risk and predictive analyses. Diabetic patients exhibited significantly higher (*p* < 0.05) urinary Cd (5.21 ± 0.83 μg/g creatinine) and Pb (0.66 ± 0.12 μg/g creatinine) levels compared with hypertensive and comorbid patients. GSH levels were markedly lower (*p* < 0.05) in diabetics than in hypertensives. Although HQs and HIs were < 1, diabetics recorded the highest HI (2.73 × 10^−1^ ± 2.27 × 10^−2^; *p* < 0.05), indicating increased chronic exposure risk. CR values for As and Cd exceeded USEPA thresholds (10^−6^), with diabetics showing the greatest risk. PCT identified Cd as the dominant contributor to HI across groups. ROC analysis showed that As CDI moderately predicted CR in diabetics (AUC = 0.604; *p* = 0.047). OLS regression revealed water source and residence as significant determinants of As and Pb exposure. Summarily, diabetic individuals demonstrated disproportionately higher heavy metal exposure and associated carcinogenic risk, largely driven by Cd. Integrating environmental biomonitoring into NCD management is essential to mitigate heavy metal–related health burdens in Cameroon.

## 1. Introduction

For over nearly 5 decades, the trajectory of heavy metal research reflects a critical transition from localized environmental assessments to an integrated concern for international food security and public health [1]. While agriculture and urbanization are key sources of heavy metal exposure, a noticeable proportion originates from the industrial and municipal wastewater. Alarmingly, nearly 80% of this wastewater is released untreated, particularly in developing nations [2]. Human exposure to heavy metals such as cadmium, lead, arsenic, mercury, nickel and copper has become a major public health concern, mainly because of their bioaccumulation, toxicity and long‐lasting nature [3].

Noncommunicable diseases (NCDs), including Type 2 diabetes mellitus and hypertension, are rising in sub‐Saharan Africa, posing a significant public health challenge [4]. Traditionally linked to lifestyle and genetic factors, there is growing recognition of environmental toxicants as hidden contributors to NCD progression [5]. Among these, heavy metals such as arsenic (As), cadmium (Cd) and lead (Pb) are of particular concern due to their ubiquity, persistence and toxicological impact on metabolic and cardiovascular systems [6].

Arsenic (As) exposure has been associated with insulin resistance and increased risk of T2DM [7], while Cd disrupts renal function and accumulates in pancreatic β‐cells, impairing glucose regulation [8]. Lead (Pb) interferes with Ca^2+^signalling and induces oxidative stress, further aggravating metabolic dysfunction [9]. In African settings, widespread use of contaminated water sources, informal mining and agricultural activities increase the risk of exposure to these heavy metals [10]. Chronic heavy metal exposure such as As, Cd and Pb exposure is associated with hepatic fibrosis, renal dysfunction, cardiovascular disease, immunosuppression and increased cancer risk (CR) characterized by excessive generation of reactive oxygen species (ROS), lipid peroxidation, mitochondrial dysfunction and inflammatory responses [11, 12]. Yet, biomonitoring studies integrating exposure, oxidative stress biomarkers and risk assessment in vulnerable patient populations remain scarce.

Diabetes and hypertensive patients may be particularly susceptible to metal toxicity due to impaired detoxification capacity and heightened oxidative stress [13]. Oxidative stress biomarkers such as reduced glutathione (GSH), thiobarbituric acid reactive substance (TBARS) and paraoxonase 1 (PON1) provide mechanistic insights into the role of redox imbalance in mediating toxicant effects [14]. However, little is known about how oxidative stress interacts with real‐world exposures in the African population with NCDs.

Health risk assessment frameworks, including chronic daily intake (CDI), hazard quotient (HQ), hazard index (HI) and CR, provide quantitative measures of exposure burden [15]. Proportion contribution trend (PCT) analysis further identifies the dominant toxicants driving risk, while receiver operating characteristics (ROC) assess the predictive capacity of biomarkers in distinguishing disease states and CR from exposure. Integrating these approaches allows a more comprehensive evaluation of cumulative risk and vulnerable groups [16].

This study presents a novel integration of biomonitoring, quantitative risk assessment and mechanistic biomarkers by simultaneously evaluating urinary heavy metal burden (As, Cd and Pb), exposure indices (EDI and CDI) and health risk metrics (HQ, HI and CR) in clinically stratified populations (diabetes, hypertensive and comorbid patients). While previous studies have typically assessed heavy metal exposure or associated health risks independently, only limited investigations have combined these standardized risk indices with oxidative stress biomarkers such as reduced GSH, TBARSs and PON1 within a clinical disease framework [17]. Furthermore, although CDI‐, HI‐ and CR‐based risk characterization is widely applied in environmental toxicology [18], their integration with proportional contribution trend (PCT) analysis and ROC modelling to assess both cumulative toxicological burden and predictive performance remains poorly explored, particularly in human biomonitoring studies [19]. This study therefore provides an important advancement linking exposure quantification, risk characterization and biological effect markers within a single analytical framework, thereby offering a more comprehensive understanding of the toxicodynamic pathways underlying cardiometabolic disease risk in sub‐Saharan African populations, where such multidimensional assessments remain scarce [20, 21].

This study assessed heavy metal exposure, including As, Cd and Pb levels and oxidative stress levels, associated with health risks in adult diabetic and hypertensive patients in Cameroon. By applying PCT and ROC analysis, this study identifies the metals most responsible for risk and the biomarkers most predictive of disease status, while ordinary least squares (OLS) regression identifies the most common sources of heavy metal exposure risk in the patients. This work addresses a critical knowledge gap in environmental determinants of NCDs in sub‐Saharan Africa and provides evidence for integrating biomonitoring into the clinical management of vulnerable populations.

## 2. Materials and Methods

### 2.1. Study Area and Location

A hospital‐based cross‐sectional study was conducted at the Diabetic and Hypertensive Unit of the Regional Hospital Bamenda, Northwest Region, Cameroon, between February and August 2025. The hospital serves as a referral centre with a diverse patient population and adequate diagnostic capacity, making it suitable for this study.

### 2.2. Study Participants and Inclusion Criteria

Eligible participants were adults ≥ 21 years with T2DM and/or hypertension. Diabetes was defined as persistent fasting blood glucose (FBS) ≥ 120 mg/dL and hypertension as persistent blood pressure ≥ 130/80 mmHg. Patients with Type 1 diabetes, secondary diabetes or a prior history of stroke, renal or hepatic failure were ineligible to participate in the study.

### 2.3. Ethical Consideration and Informed Consent

Ethical clearance and administrative authorization were obtained from the Regional Delegation of Public Health, Bamenda (Ref No.: 2025/03/52/CERSH‐NW), respectively. Written informed consent was obtained from all participants, and confidentiality was maintained throughout the study.

### 2.4. Sample Size and Data Collection

The estimated population who visited the diabetic and hypertensive clinic of the Regional Hospital Bamenda was about 212 combined diabetic and hypertensive patients, from which the sample size was calculated using the formula for sample size calculations as described by Yamane [22], *n* = *N*/(1 + Ne^2^), where *n* and N represent the corrected sample and population size (212 patients), respectively. The margin of error was *e* = 0.05, giving an estimated population of 138. The final recruited participant population comprised 178 patients distributed into 39 diabetic patients, 78 hypertensive patients and 61 diabetic and hypertensive patients.

### 2.5. Data Collection

Sociodemographic, lifestyle, anthropometric and the possible sources of environmental exposure data were collected using well‐structured questionnaires. Urine samples were collected in a 60 mL polyethylene container, frozen at −20 °C, until heavy metal analysis. Blood was collected via venipuncture, following standard procedures, and centrifuged at 3000 rpm for 10 min, and the serum obtained was used for oxidative stress analyses.

### 2.6. Urine Creatinine and Heavy Metal Analysis

Urine creatinine was assessed using the Jaffe kinetic method Toora et al. [23], with a commercially available kit (Abbott Laboratories, USA). Briefly, 50 μL of 10 times diluted urine sample was combined with 1 mL of working reagent and incubated for 3 min at 37°C. Then, 200 μL of working reagent was added and absorbance was read at 510 nm using a spectrophotometer (UV^−1^900i Spectrophotometer, Shimadzu Corporation, Tokyo, Japan).

For heavy metal analysis, 2 mL of 65% nitric acid (HNO_3_) was added to 5 mL of the urine sample to prevent heavy metal degradation and the pH was adjusted close to 2. Then, the mixture was submitted to acid digestion by addition 3 mL of HNO_3_ and 1 mL of 37% HCl and incubated at 100 °C for 30 min. This digestion liberates heavy metals bound to proteins and other organic molecules. After cooling, the concentration of acid in the medium was diluted to 1% with ultrapure water and Pb, As and Cd levels were analysed at their respective wavelength (283.3, 193.7 and 228.8 nm, respectively) against the blank using atomic absorption (ASC^-7^800 autosampler, Shimadzu Corporation, Tokyo, Japan) and normalized to urine creatinine [24]. Standard creatinine solution (2 mg/dL) was used in the quantification of urine creatinine.

### 2.7. Oxidative Stress Analyses

Serum PON1 activity was determined according to the methods described by Joseet al. [25], with modifications. Briefly, a 1:3 dilution of serum was mixed in 50 mM Tris‐HCl buffer pH 8.0, containing 1 mM CaCl_2_.2 H_2_O [3.0285g Tris+10 mL 1M HCl +0.0735g CaCl_2_.2 H_2_O in 500 mL]. Then, 28 µL of diluted serum was pipetted into 400 μL of 50 mM buffer. The reaction was initiated by adding 100 µL of 6.3 mg/mL 4‐phenylacetate as the substrate. An increase in absorbance was measured at 405 nm every 10‐s interval for 210s, using a spectrophotometer. The PON1 activity was expressed in units per litre of serum, with 1 unit per litter representing the number of micromoles of substrate converted into phenol per minute by PON1 enzyme in serum.

TBARS levels were determined using the TBA method [26]. Briefly, 100 µL of serum was mixed with 1 mL of a working solution containing 15% (w/v) trichloroacetic acid, 0.38% (w/v) TBA and 0.25N of hydrochloric acid. The mixture was heated at 100 °C for 30 min and then centrifuged (2500g, 5 min), and the absorbance was measured at 532 nm using a UV–visible spectrophotometer. Serum TBARS concentration was calculated using the molar extinction coefficient of 155 mM^−1^ cm^−1^ and read at 530 nm.

GSH levels were quantified using the Ellman method [27], with modifications. Briefly, 20 μL of the participant’s serum was mixed with 750 µL of phosphate buffer pH 6.5, and 750 µL of 1 mM DNTB was added. The mixture was incubated in the dark at room temperature for 60 min. The mixture was transferred into a 96‐well plate, and absorbance was read at 405 nm using a microplate reader (Elabscience, Houston, Texas, USA). The GSH levels in samples were deduced using a standard of GSH (concentrations 0–1.5 mM) prepared alongside the analysed samples.

### 2.8. Risk Assessment Methods

Health risk assessment was conducted following the United States Environmental Protection Agency (USEPA) guidelines for exposure to toxic substances through ingestion, inhalation and dermal contact pathways [28]. Given the biological monitoring design of this study, urinary heavy metal levels (As, Cd and Pb) were normalized to creatinine and used as biomarkers of exposure, which is consistent with World Health Organization (WHO) and the Agency for Toxic Substances and Disease Registry (ATSDR) recommendations for internal dose assessment [29, 30].

### 2.9. Estimated Daily Intake (EDI) and CDI

EDI (mg/kg/day) and CDI (mg/kg/day) were calculated to estimate exposure intensity and long‐term risk from heavy metals using standard equations defined by USEPA [15].

(1) EDI=C X Scr24hrsBW×f,

(2) CDI=C×IR X EF X ED Bw X AT,

where C, SCr_24hrs_, EF, ED, BW, AT and *f* are the concentration of heavy metal (mg/kg, creatinine‐normalized for urine), total urinary creatinine excreted within 24 h [15], exposure frequency (365 days/year), exposure duration (15 years, based on average residency and disease history), body weight (kg), averaging time (non‐CR = ED × 365 days; carcinogenic risk = lifetime, i.e., 70 years × 365 days) and urinary excretion factor of heavy metals (As = 60% (0.6), Cd = 0.005% (0.00005), Pb = 5.4% (0.054)), respectively.

### 2.10. HQ

The HQ was used to determine the potential for noncancer health effects from exposure to heavy metals [29]. The HQ was calculated as a ratio of CDI to the reference dose (RfD, mg/kg/day) established by USEPA.

(3) H Q= CDIRfD,

where CDI is a chronic daily intake (mg/kg/day) and RfD is the reference dose of heavy metal (mg/kg/day). RfD was as follows: As = 3.0 × 10^−4^ mg/kg/day, Cd = 1.0 × 10^−3^ mg/kg/day and Pb = 3.5 × 10^−3^ mg/kg/day [31]. An HQ < 1 indicates the negligible risk, while HQ > 1 suggests the potential noncarcinogenic effects [32].

### 2.11. HI

The HI was derived as the sum of HQ across all metals for each participant group:

(4) H I=∑HQ,

where ∑*H* *Q* represents the sum of HQ of all toxicants. An HI > 1 indicates the potential for combined adverse effects from multiple exposures [32].

### 2.12. CR Assessment

Lifetime CR was estimated using cancer slope factors (CSFs) from USEPA’s Integrated Risk Information System (IRIS): As = 1.5 (mg/kg/day)^−1^ and Cd = 0.38 (mg/kg/day)^−1^, while Pb is not considered carcinogenic.

(5) Cancer risk=CDI×CSF,

where CDI and CSF represent the chronic daily intake (mg/kg/day) and CSF of the heavy metal, respectively.

### 2.13. Statistical Analysis

Data were analysed using IBM SPSS v25. Continuous variables were expressed as mean ± SEM. Normality was testing using skewness and kurtosis and ANOVA with Duncan’s multiple range post hoc test was used for group comparisons. Pearson’s correlation was used to assess the relationship between heavy metal exposure, oxidative stress and health risk in the different groups of patients to determine which patients are most susceptible to health risks, while PCT analysis was used to evaluate the contribution of heavy metal exposure to health risk (HI). The ROC analysis was used as a predictive determinant for the impact of heavy metal exposure on disease condition and CR in various disease groups and other sociodemographic factors of health risk while OLS regression identified the most common sources of heavy metal exposure risk in the patients.

## 3. Results

### 3.1. Demographic Characteristics of Participants

A total of 178 participants enrolled, comprising 21.9% diabetics, 43.8% hypertensive and 34.3% with both disease conditions. Females predominated, with a proportion of 65.2%. The majority of participants (44.9%) were aged 55–69 years, while only 1% were ≥ 85 years old. Educational levels varied widely, with a notable 15.5% of females having no formal education. Most participants resided in urban areas (60.7%), while rural dwellers constituted 14.6%. Occupationally, farmers (40.4%) and traders (27%) were the largest groups. Tap water (33.1%) and borehole (27.0%) were the main drinking water sources, whereas well water and streams represented minor but important water sources (Table 1).

**TABLE 1:** Demographic characteristics of participants.

	Variables	Male (*n*, %)	Female (*n*, %)	Total (*n*, %)
Disease status	Diabetic patients	15 (24.2)	24 (20.7)	39 (21.9)
	Hypertensive patients	22 (35.5)	39 (33.6)	61 (34.3)
	Diabetic and hypertensive patients	25 (40.3)	53 (45.7)	78 (43.8)
	Total	62 (100.0)	116 (100.0)	178 (100.0)

Education	No education	0 (0.0)	18 (15.5)	18 (10.1)
	Primary	27 (43.5)	43 (37.1)	70 (39.3)
	Secondary	23 (37.1)	36 (31.1)	59 (33.1)
	University	12 (19.4)	19 (31.0)	31 (17.4)
	Total	62 (100.0)	116 (100.0)	178 (100.0)

Occupation	Farmer	10 (16.1)	62 (53.4)	72 (40.4)
	commercial/vendor/trader	20 (32.3)	27 (23.3)	47 (26.4)
	Teacher	11 (17.7)	16 (13.8)	27 (15.2)
	Driver	5 (8.1)	0 (0.0)	5 (2.8)
	Finance officers	2 (3.2)	4 (3.4)	6 (3.4)
	Engineer	10 (16.1)	1 (0.9)	11 (6.2)
	Medical personnel	4 (6.5)	6 (5.2)	10 (5.6)
	Total	62 (100.0)	116 (100.0)	178 (100.0)

Residence	Rural	11 (17.7)	15 (12.9)	26 (14.6)
	Suburban	10 (16.1)	34 (29.3)	44 (24.7)
	Urban	41 (66.1)	67 (57.8)	108 (60.7)
	Total	62 (100.0)	116 (100.0)	178 (100.0)

Water source	Bottled water	16 (25.8)	19 (16.4)	35 (19.7)
	Bottled water and forage	4 (6.5)	7 (6.0)	11 (6.2)
	Bottled water and tap water	1 (1.6)	4 (3.4)	5 (2.8)
	Borehole	17 (27.4)	31 (26.7)	48 (27.0)
	Stream	3 (4.8)	12 (10.3)	15 (8.4)
	Tap water	20 (32.3)	39 (33.6)	59 (33.1)
	Well water	1 (1.6)	4 (3.4)	5 (2.8)
	Total	62 (100.0)	116 (100.0)	178 (100.0)

*Note:* Values on the table represent the absolute frequencies (no brackets) and frequency distribution (in brackets and italicized) of the demographic of respondents represented as percentages.

### 3.2. Heavy Metal Exposure and Oxidative Stress Levels Among the Population

Diabetic patients exhibited higher urinary As (1.41 ± 0.29 vs. 0.96 ± 0.17 µg/g creatinine) and significantly higher Cd levels (5.21 ± 0.83 µg/g creatinine vs. 3.97 ± 0.60 µg/g creatinine, *p* < 0.05) when compared to hypertensive and comorbid cases (4.03 ± 0.54 µg/g creatinine). Lead levels were also elevated in diabetics (0.66 ± 0.12 μg/g creatinine) relative to hypertensives (0.43 ± 0.07 μg/g creatinine) and comorbid patients (0.40 ± 0.07 μg/g creatinine, *p* < 0.05). Heavy metal exposure and oxidative stress biomarkers showed no significant differences between males and females (*p* > 0.05) (Table 2).

**TABLE 2:** Heavy metal and oxidative stress levels among the population.

	Variables	As (μg/g creatinine)	Cd (μg/g creatinine)	Pb (μg/g creatinine)
Disease status	Diabetics (*n* = 39)	1.41 ± 0.29^a^ (0.62)	5.21 ± 0.83^b^ (2.97)	0.66 ± 0.12^b^ (0.47)
	Hypertensives (*n* = 61)	1.08 ± 0.18^a^ (0.65)	3.97 ± 0.60^a^ (2.11)	0.43 ± 0.07^a^ (0.20)
	Diabetics and hypertensives (*n* = 78)	0.96 ± 0.17^a^ (0.31)	4.03 ± 0.54^ab^ (2.46)	0.40 ± 0.07^a^ (0.26)

Gender	Male (*n* = 62)	1.07 ± 0.19^a^ (0.50)	4.23 ± 0.61^a^ (2.39)	0.45 ± 0.06^a^ (0.28)
	Female (*n* = 116)	1.12 ± 0.15^a^ (0.54)	4.28 ± 0.45^a^ (2.44)	0.48 ± 0.06^a^ (0.23)

Residents	Rural (*n* = 26)	1.70 ± 0.38^b^ (1.25)	5.25 ± 0.91^b^ (3.66)	0.71 ± 0.20^b^ (0.46)
	Suburban (*n* = 44)	0.81 ± 0.13^a^ (3.94)	4.06 ± 0.52^a^ (3.22)	0.46 ± 0.08^ab^ (0.27)
	Urban (*n* – 108)	1.08 ± 0.16^ab^ (0.44)	4.12 ± 0.52^a^ (2.16)	0.42 ± 0.05^a^ (0.23)

Type of drinking water	Bottled water (*n* = 35)	1.20 ± 0.26^a^ (0.62)	4.63 ± 0.95^ab^ (2.83)	0.43 ± 0.06^a^ (0.29)
	Bottled water and for age (*n* = 11)	0.42 ± 0.11^a^ (0.27)	3.59 ± 1.66^ab^ (1.84)	0.14 ± 0.02^a^ (0.13)
	Bottled and tap water (*n* = 3)	1.11 ± 0.60^a^ (0.41)	1.72 ± 0.57^a^ (1.26)	0.14 ± 0.04^a^ (0.12)
	For age (*n* = 48)	1.14 ± 0.24^a^ (0.41)	2.98 ± 0.32^ab^ (2.10)	0.41 ± 0.06^a^ (0.21)
	Stream (*n* = 15)	0.97 ± 0.16^a^ (0.94)	6.46 ± 1.35^b^ (4.85)	0.63 ± 0.14^a^ (0.46)
	Tap water (*n* = 59)	1.11 ± 0.22^a^ (0.53)	4.81 ± 0.74^ab^ (2.38)	0.48 ± 0.08^a^ (0.26)
	Wells (*n* = 5)	1.84 ± 1.37^a^ (0.74)	5.14 ± 1.69^ab^ (7.00)	1.72 ± 0.84^b^ (1.50)

*Note:* Values represent mean ± SEM (median) with superscripts a, b representing the statistical difference vertically in a column, significant at *p* < 0.05, ANOVA, Duncan multiple comparison post hoc test carried at 95% confidence interval.

Arsenic levels were the highest in the 24–39‐year group (1.56 ± 0.81 μg/g creatinine) and those aged 55–69 years (1.36 ± 0.21 μg/g creatinine), but not statistically significant. Cd exposure peaked in the ≥ 85 group (8.94 ± 3.11 μg/g creatinine), almost double that of other groups. Pb remained stable across ages (∼0.38–0.52 μg/g creatinine) (Supporting Table 1). Based on the geographical location, rural residents exhibited significantly higher levels of As (1.70 ± 0.38 μg/g creatinine, *p* < 0.05) and Cd (5.25 ± 0.91 μg/g creatinine, *p* < 0.05) compared with suburban and urban dwellers. A similar trend was also observed in Pb with rural residents (0.71 ± 0.20 μg/g creatinine, *p* < 0.05) displaying higher levels than urban counterparts (Table 2).

Stream water users had elevated Cd exposure (6.46 ± 1.35 μg/g creatinine, *p* < 0.05), while well users had markedly higher Pb (1.72 ± 0.84 μg/g creatinine, *p* < 0.05) as compared to bottled and tap water, and other water sources, respectively (Table 2).

### 3.3. Heavy Metal Exposure at Various Percentiles

The quantified heavy metal exposure demonstrated a highly right skewed distribution for both arsenic (As) and cadmium (Cd) in the population, where the median (P_50_) was significantly lower than the mean for both metals, indicating the presence of a few highly exposed individuals (Table 3). For As, the typical exposure level (P_50_) was 0.53 μg/g creatinine, with the lowest 5% of the population below P_5_ (0.05 μg/g creatinine) and 75% below 1.22 μg/g creatinine. For Cd, concentrations were generally much higher than As, with a median (P_50_) of 2.41 μg/g creatinine, ranging from a P_5_ of 0.59 μg/g creatinine to a P_75_ of 5.18 μg/g creatinine. Critically, the maximum exposure (P_100_) showed extreme values for both metals (As and Cd), reaching 7.8 μg/g creatinine for As and an alarming 30.20 μg/g creatinine for Cd, highlighting a significant toxicological risk for the most highly exposed individuals in the cohort. Lead (Pb) showed the lowest central tendencies, with a mean of 0.47 μg/g and a median (P_50_) of 0.25 μg/g creatinine where the bottom 5% was below P_5_ (0.05 μg/g creatinine) and the upper quartile (P_75_) was below 0.59 μg/g creatinine, but still reached a maximum (P_100_) of 4.69 μg/g creatinine), confirming that high exposure outliers are a concern across all three measured heavy metals.

**TABLE 3:** Urinary heavy metal exposure (μg/g creatinine) at different percentiles across study groups.

	Heavy metal	Mean ± SEM	p5	p25	p50 (median)	p75	p100 (max)
Overall population	As (μg/g creatinine)	1.10 ± 0.12	0.05	0.18	0.53	1.22	7.78
	Cd (μg/g creatinine)	4.27 ± 0.36	0.59	1.29	2.41	5.18	30.20
	Pb (μg/creatinine)	0.47 ± 0.05	0.05	0.13	0.25	0.59	4.69

Diabetes	As (μg/g creatinine)	1.41 ± 0.25	0.11	0.39	0.62	1.34	6.46
	Cd (μg/g creatinine)	5.21 ± 0.83	0.64	1.49	2.97	6.75	22.03
	Pb (μg/creatinine)	0.66 ± 0.12	0.05	0.13	0.47	0.83	3.06

Hypertension	As (μg/g creatinine)	1.27 ± 0.23	0.07	0.24	0.53	1.22	7.78
	Cd (μg/g creatinine)	4.95 ± 0.65	0.64	1.28	2.93	6.56	22.03
	Pb (μg/creatinine)	0.55 ± 0.08	0.05	0.13	0.31	0.71	3.06

Diabetes and hypertension	As (μg/g creatinine)	1.34 ± 0.21	0.05	0.24	0.58	1.29	7.78
	Cd (μg/g creatinine)	5.20 ± 0.64	0.59	1.26	3.06	6.81	30.20
	Pb (μg/creatinine)	0.57 ± 0.09	0.04	0.12	0.30	0.70	4.69

*Note:* Values represent the mean values at different percentiles p5, p25, p50, p75 and p100 of patient exposure to heavy metals.

The diabetes group exhibits mean urinary heavy metal concentrations of 1.41 ± 0.25 μg/g creatinine for As, 5.21 ± 0.83 μg/g creatinine for Cd and 0.66 ± 0.12 μg/g creatinine for Pb. Consistent with the overall data trend, Cd shows the highest average concentration, followed by As and then Ld. The distribution for Cd is wide, ranging from a P_5_ of 0.64 to a maximum (P_100_) of 22.03 μg/g creatinine, indicating that a small fraction of diabetic patients have significantly higher exposure or retention of this metal. In the hypertension group, the mean heavy metal levels were 1.27 ± 0.23, 4.95 ± 0.65 and 0.55 ± 0.08 μg/g creatinine for As, Cd and Pb, respectively. These mean values were slightly lower than those observed in the diabetes group for all three metals, although the P_75_ values for As (1.22) and Cd (6.56) were close to those in the diabetes group. The highest recorded value (P_100_) for Cd, 22.03 μg/g creatinine, was identical to the maximum found in the diabetes group. The group with both conditions, diabetes and hypertension, showed mean concentrations of 1.34 ± 0.21, 5.20 ± 0.64 and 0.57 ± 0.09 μg/g creatinine for As, Cd and Pb, respectively. These mean values were highly comparable to the individual disease groups. However, this group exhibited the absolute highest maximum concentration (P_100_) for Cd at 30.20 μg/g creatinine, suggesting that the combination of these two diseases may be associated with the most severe outlier exposures or retention issues for this chemical. Lead had the lowest mean concentration and the lowest maximum (4.69 μg/g creatinine) among the three metals for this group.

### 3.4. Predictive Capacity of Sociodemographic Characteristics for Heavy Metal Exposure

Sociodemographic characteristics (*R* ^2^ = 0.031, *p* = 0.598) showed very weak predictive capacity for heavy metal exposure. The residence effect marginally contributed to As exposure levels (*B* = −0.189, *t* = −1.143, 95% CI = −0.516–0.138), while all other variables including age group (*p* = 0.639), gender (*p* = 0.904) and occupation (*p* = 0.269) did not show significant contributions to exposure. Source of water attained statistical significance for As exposure (*B* = 0.006, *t* = 0.094, *p* < 0.025, 95% CI = −0.118 – 0.130), indicating its significant contribution to exposure trends from the regression analysis. The Pb model showed the highest but still modest fit (*R* ^2^ = 0.077, Adjusted *R* ^2^ = 0.039, *p* = 0.054). Residence and water source displayed significant effects or contributions to Pb exposure just as in As exposure (*B* = −0.113, *p* = 0.049, 95% CI = −0.239 to 0.013; *B* = 0.043, *p* = 0.045, 95% CI = −0.005 to 0.091, respectively). Other predictors, including gender (*p* = 0.915), occupation (*p* = 0.422) and age group (*p* = 0.911), were not significant showing no direct marked contributions to exposure frequencies (Table 4).

**TABLE 4:** Predictive capacity for sociodemographic characteristics on heavy metal exposure.

Dependent variable	Predictor	B (coefficient)	Std. error	t value	*p* value	95% CI (lower–upper)	*R* ^2^
Arsenic (As)	Constant	1.678	0.873	1.923	0.056	−0.045–3.402	0.031
	Age groups	0.069	0.148	0.470	0.639	−0.222–0.361	
	Gender	0.030	0.250	0.121	0.904	−0.463–0.524	
	Occupation	0.066	0.059	1.109	0.269	−0.051–0.182	
	Residence	−0.189	0.166	−1.143	0.255	−0.516–0.138	
	Type of water	0.006	0.063	0.094	0.025	−0.118–0.130	

Cadmium (Cd)	Constant	5.916	2.726	2.170	0.031^∗^	0.535–11.296	0.021
	Age groups	0.281	0.462	0.608	0.544	−0.631–1.192	
	Gender	0.070	0.780	0.090	0.928	−1.471–1.611	
	Occupation	−0.060	0.185	−0.324	0.747	−0.425–0.305	
	Residence	−0.422	0.517	−0.817	0.415	−1.443–0.598	
	Type of water	0.121	0.197	0.613	0.041	−0.268–0.509	

Lead (Pb)	Constant	0.837	0.336	2.488	0.014^∗^	0.173–1.501	0.077
	Age groups	−0.006	0.057	−0.112	0.911	−0.119–0.106	
	Gender	0.010	0.096	0.107	0.915	−0.180–0.200	
	Occupation	0.018	0.023	0.804	0.422	−0.027–0.063	
	Residence	−0.113	0.064	−1.765	0.049	−0.239–0.013	
	Type of water	0.043	0.024	1.789	0.045	−0.005–0.091	

*Note:* Ordinary least squares regression significant at *p* < 0.05. *B* = coefficient, *t* value = test value, CI 95% = confidence interval 95%.

^∗^Significant model fit for regression statistics.

Residual distributions for all metals approximated normal curves with mean ≈ 0 and SD ≈ 1, confirming the normality of residuals required for valid OLS inference. The histograms exhibited approximately normal distributions centred near zero, validating the assumption of normally distributed residuals essential for parametric inference. Pb showed the closest conformity to normality (Supporting Figure 1). Residuals were evenly distributed around zero for As, Cd and Pb, indicating homoscedasticity and absence of bias in model fitting. Each plot (As, Cd, Pb) demonstrates the random dispersion of residuals about zero, confirming homoscedasticity and absence of systematic error. The uniform spread indicated that variance in predicted heavy metal concentrations remained constant across fitted values (Supporting Figure 2). Observed and expected cumulative probabilities aligned closely along the diagonal line, demonstrating normal residual patterns with minimal skewness. Observed cumulative probabilities align closely with expected normal quantiles for all models, confirming the appropriateness of linear modelling. Minor deviations at the tails suggest slight skewness without violating model validity (Supporting Figure 3).

### 3.5. Oxidative Stress

Diabetics showed significantly reduced GSH (38.23 ± 3.27 mg/dL, *p* < 0.05) and TBARS (1.61 ± 0.11 mg/dL, *p* < 0.05), suggesting impaired oxidative defence compared to hypertensive and comorbid groups (Table 4). However, GSH, TBARS and PON1 were broadly similar across age (Supporting Table 1) groups but differed in well users, with TBARS significantly higher (4.73 ± 0.75 mg/dL vs. 1.94 ± 0.24 mg/dL, *p* < 0.05) and lower PON1 (0.08 ± 0.05 U/L vs 0.23 ± 0.02 U/L, *p* < 0.05) (Table 5).

**TABLE 5:** Oxidative stress biomarkers among patients.

	Variables	Oxidative stress biomarkers		
		GSH (mg/dL)	TBARS (mg/dL)	PON1 (U/L)
Disease status	Diabetics (*n* = 39)	38.23 ± 3.27^a^	1.61 ± 0.11^a^	0.23 ± 0.21^a^
	Hypertensives (*n* = 61)	57.75 ± 4.71^b^	2.91 ± 0.15^b^	0.20 ± 0.02^a^
	Diabetics and hypertensives (*n* = 78)	53.43 ± 3.01b	2.75 ± 0.14^b^	0.20 ± 0.02^a^

Gender	Male (*n* = 62)	52.47 ± 4.14^a^	2.65 ± 0.55^a^	0.20 ± 0.07^a^
	Female (*n* = 116)	51.11 ± 2.69^a^	2.50 ± 0.11^a^	0.21 ± 0.04^a^

Residents	Rural (*n* = 26)	51.75 ± 4.92^a^	2.71 ± 0.26^a^	0.20 ± 0.03^a^
	Suburban (*n* = 44)	51.65 ± 3.96^a^	2.68 ± 0.22^a^	0.22 ± 0.02^a^
	Urban (*n* – 108)	51.51 ± 3.16^a^	2.47 ± 0.11^a^	0.20 ± 0.01^a^

Type of drinking water	Bottled water (*n* = 35)	55.17 ± 6.17^a^	2.45 ± 0.21^a^	0.22 ± 0.02^b^
	Bottled water and forage (*n* = 11)	54.65 ± 7.74^a^	2.68 ± 0.45^a^	0.16 ± 0.04^ab^
	Bottled and tap water (*n* = 3)	44.70 ± 10.64^a^	1.94 ± 0.24^a^	0.21 ± 0.08^ab^
	Forage (*n* = 48)	53.90 ± 4.97^a^	2.62 ± 0.16^a^	0.23 ± 0.02^b^
	Stream (*n* = 15)	48.51 ± 5.63^a^	2.36 ± 0.28^a^	0.15 ± 0.04^ab^
	Tap water (*n* = 59)	47.56 ± 2.95^a^	2.45 ± 0.15^a^	0.20 ± 0.02^ab^
	Wells (*n* = 5)	60.98 ± 24.57^a^	4.73 ± 0.75^b^	0.08 ± 0.05^a^
	Tot al(*n* = 178)	51.58 ± 2.26	2.56 ± 0.92	0.20 ± 0.01

*Note:* Values represent mean ± SEM with superscripts a, b representing the statistical difference vertically in a column, significant at *p* < 0.05, ANOVA, Duncan multiple comparison post hoc test carried at 95% confidence interval.

### 3.6. Risk Assessment

Across all groups, EDI and CDI for As, Cd and Pb remained below their reference levels, which are 3.0 × 10^−4^ mg/kg/day, 1.0 × 10^−3^ mg/kg/day and 3.5 × 10^−3^ mg/kg/day, respectively. However, diabetic patients consistently had higher CDI (As: 1.83 × 10^−5^± 4.33 × 10^−6^ mg/kg/day, Cd: 5.07 × 10^−5^ ± 9.00 × 10^−6^ mg/kg/day and Pb: 8.03 × 10^−6^ ± 1.44 × 10^−6^ mg/kg/day, *p* < 0.05) and EDI (As: 1.83 × 10^−5^ ± 4.33 × 10, Cd: 6.34 × 10^−5^ ± 1.12 × 10^−5^ and Pb: 8.03 × 10^−6^ ± 1.44 × 10^−6^) values higher than hypertensive and comorbid patients reflecting greater vulnerability to health issues (Table 6).

**TABLE 6:** Chronic daily intake and estimated daily intake assessment.

	Patient status	CDI (mg/kg/day)			EDI (mg/kg/day)		
		As	Cd	Pb	As	Cd	Pb
Daily risk assessment by gender	Diabetics (*n* = 39)	1.83 × 10^−5^ ± 4.33 × 10^−6b^	5.07 × 10^−5^ ± 9.00 × 10^−6b^	8.03 × 10^−6^ ± 1.44 × 10^−6b^	1.83 × 10^−5^ ± 4.33 × 10^−6b^	6.34 × 10^−5^ ± 1.12 × 10^−5b^	8.03 × 10^−6^ ± 1.44 × 10^−6b^
	Hypertensives (*n* = 61)	1.22 × 10^−5^ ± 2.03 × 10^−6ab^	3.45 × 10^−5^ ± 5.02 × 10^−6a^	4.82 × 10^−6^ ± 8.33 × 10^−7a^	1.22 × 10^−5^ ± 2.03 × 10^−6ab^	4.31 × 10^−5^ ± 6.29 × 10^−6a^	4.82 × 10^−6^ ± 8.33 × 10^−7a^
	Diabetics and hypertensives (*n* = 78)	1.01 × 10^−5^ ± 1.29 × 10^−6a^	3.29 × 10^−5^ ± 4.12 × 10^−6a^	4.14 × 10^−6^ ± 6.36 × 10^−7a^	1.10 × 10^−5^ ± 1.69 × 10^−6a^	4.11 × 10^−5^ ± 5.15 × 10^−6a^	4.14 × 10^−6^ ± 6.36 × 10^−7a^
	Tot al (178)	1.26 × 10^−5^ ± 1.39 × 10^−6^	3.73 × 10^−5^ ± 3.19 × 10^−6^	5.21‐ × 10^−6^ ± 5.16 × 10^−7^	1.26 × 10^−5^ ± 1.39 × 10^−6^	4.66 × 10^−5^ ± 3.99 × 10^−6^	5.21 × 10^−6^ ± 5.16 × 10^−7^

Gender	Male (*n* = 62)	1.18 × 10^−5^ ± 1.93 × 10^−6a^	3.61 × 10^−5^ ± 5.00 × 10^−6a^	5.03 × 10^−6^ ± 7.70 × 10^−7a^	1.18 × 10^−5^ ± 1.92 × 10^−6a^	4.51 × 10^−5^ ± 6.24 × 10^−6a^	5.03 × 10^−6^ ± 7.70 × 10^−7a^
	Fem ale (*n* = 116)	1.29 × 10^−5^ ± 1.87 × 10^−6a^	3.78 × 10^−5^ ± 4.08 × 10^−6a^	5.28 × 10^−6^ ± 6.74 × 10^−7a^	1.30 × 10^−5^ ± 1.86 × 10^−6a^	4.72 × 10^−5^ ± 5.10 × 10^−6a^	5.28 × 10^−6^ ± 6.74 × 10^−7a^
	Total (178)	1.26 × 10^−5^ ± 1.39 × 10^−6^	3.73 × 10^−5^ ± 3.19 × 10^−6^	5.21‐ × 10^−6^ ± 5.16 × 10^−7^	1.26 × 10^−5^ ± 1.39 × 10^−6^	4.66 × 10^−5^ ± 3.99 × 10^−6^	5.21 × 10^−6^ ± 5.16 × 10^−7^

Residents	Rural (*n* = 26)	1.98 × 10^−5^ ± 4.36 × 10^−6b^	4.83 × 10^−5^ ± 4.36 × 10^−6a^	8.04 × 10^−6^ ± 2.05 × 10^−6b^	1.97 × 10^−5^ ± 4.37 × 10^−6b^	6.04 × 10^−5^ ± 1.11 × 10^−5a^	8.04 × 10^−6^ ± 9.92 × 10^−7b^
	Suburban (*n* = 44)	9.14 × 10^−6^ ± 1.56 × 10^−6a^	3.54 × 10^−5^ ± 1.56 × 10^−6a^	5.07 × 10^−6^ ± 9.92 × 10^−7a^	9.05 × 10^−6^ ± 1.56 × 10^−6a^	4.42 × 10^−5^ ± 5.57 × 10^−6a^	5.07 × 10^−6^ ± 5.47 × 10^−7ab^
	Urban (*n* – 108)	1.23 × 10^−5^ ± 1.90 × 10^−6ab^	3.53 × 10^−5^ ± 1.90 × 10^−6a^	4.56 × 10^−6^ ± 5.47 × 10^−7a^	1.22 × 10^−5^ ± 1.89 × 10^−6ab^	4.41 × 10^−5^ ± 5.50 × 10^−5a^	4.56 × 10^−6^ ± 5.47 × 10^−7a^
	Total (*n* = 178)	1.25 × 10^−5^ ± 1.39 × 10^−6^	3.72 × 10^−5^ ± 1.39 × 10^−6^	5.19 × 10^−6^ ± 5.13 × 10^−7^	1.25 × 10^−5^ ± 1.38 × 10^−6^	4.65 × 10^−5^ ± 2.05 × 10^−6^	4.19 × 10^−6^ ± 5.13 × 10^−7^

Drinking water	Bottled water (*n* = 35)	1.49 × 10^−5^ ± 3.99 × 10^−6a^	4.18 × 10^−5^ ± 8.82 × 10^−6ab^	4.69 × 10^−6^ ± 6.79 × 10^−7a^	1.49 × 10^−5^ ± 3.97 × 10^−6a^	5.22 × 10^−5^ ± 1.10 × 10^−5ab^	4.69 × 10^−6^ ± 6.79 × 10^−7a^
	Bottled water and forage (*n* = 11)	4.45 × 10^−6^ ± 1.27 × 10^−6a^	2.68 × 10^−5^ ± 1.09 × 10^−5ab^	1.42 × 10^−6^ ± 2.29 × 10^−7a^	4.60 × 10^−6^ ± 1.28 × 10^−6a^	3.35 × 10^−5^ ± 1.36 × 10^−5ab^	1.42 × 10^−6^ ± 2.29 × 10^−7a^
	Bottled and tap water (*n* = 3)	1.26 × 10^−5^ ± 6.91 × 10^−6a^	1.58 × 10^−5^ ± 5.64 × 10^−6a^	1.53 × 10^−6^ ± 4.63 × 10^−7a^	1.27 × 10^−5^ ± 6.85 × 10^−6a^	1.97 × 10^−5^ ± 7.06 × 10^−6a^	1.53 × 10^−6^ ± 4.63 × 10^−7a^
	Forage (*n* = 48)	1.25 × 10^−5^ ± 2.75 × 10^−6a^	2.53 × 10^−5^ ± 2.83 × 10^−6ab^	4.46 × 10^−6^ ± 7.38 × 10^−7a^	1.26 × 10^−5^ ± 2.75 × 10^−6a^	3.17 × 10^−5^ ± 3.54 × 10^−6ab^	4.46 × 10^−6^ ± 7.38 × 10^−7a^
	Stream (*n* = 15)	1.13 × 10^−5^ ± 1.97 × 10^−6a^	5.77 × 10^−5^ ± 1.22 × 10^−5b^	7.22 × 10^−6^ ± 1.63 × 10^−6a^	1.12 × 10^−5^ ± 1.99 × 10^−6a^	7.21 × 10^−5^ ± 1.52 × 10^−5b^	7.22 × 10^−6^ ± 1.63 × 10^−6a^
	Tap water (*n* = 59)	1.25 × 10^−5^ ± 2.32 × 10^−6a^	4.23 × 10^−5^ ± 6.36 × 10^−6ab^	5.59 × 10^−6^ ± 1.05 × 10^−6a^	1.25 × 10^−5^ ± 2.33 × 10^−6a^	5.29 × 10^−5^ ± 7.95 × 10^−6ab^	5.59 × 10^−6^ ± 1.04 × 10^−6a^
	Wells (*n* = 5)	1.76 × 10^−5^ ± 1.22 × 10^−6a^	4.24 × 10^−5^ ± 1.41 × 10^−5ab^	1.69 × 10^−5^ ± 7.76 × 10^−6b^	1.74 × 10^−5^ ± 1.24 × 10^−5a^	5.30 × 10^−5^ ± 1.76 × 10^−5ab^	1.69 × 10^−5^ ± 7.76 × 10^−6b^
	Tot al (*n* = 178)	1.25 × 10^−5^ ± 1.39 × 10^−6^	3.72 × 10^−5^ ± 3.17 × 10^−6^	5.19 × 10^−6^ ± 5.13 × 10^−7^	1.25 × 10^−5^ ± 1.38 × 10^−6^	4.65 × 10^−5^ ± 3.96 × 10^−6^	5.19 × 10^−6^ ± 5.13 × 10^−7^

*Note:* Values represent mean ± SEM with superscripts a, b representing the statistical difference vertically in a column, significant at *p* < 0.05, ANOVA, Duncan multiple comparison post hoc test carried at 95% confidence interval.

### 3.7. HI and CR Assessment

#### 3.7.1. HQ

The HQ for As was the highest in diabetic patients (6.09 × 10^−2^ ± 1.44 × 10^−2^ mg/kg/day, *p* < 0.05) as compared to that of hypertensive patients (4.10 × 10^−2^ ± 6.78 × 10^−3^ mg/kg/day) and comorbid patients (diabetes and hypertension) (3.07 × 10^−2^ ± 5.65 × 10^−3^ mg/kg/day) (Table 7).

**TABLE 7:** Health risk assessment parameters (hazard quotients, hazard index and cancer risk).

	Variables	Health hazard quotient (HQ)			Hazard index (HI)	Cancer risk (CR)		
		As	Cd	Pb	Sum (HQ) (HI)	As ((mg/kg/day)^−1^)	Cd ((mg/kg/day)^−1^)	Pb ((mg/kg/day)^−1^)
Disease status	Diabetics (*n* = 39)	6.09 × 10^−2^ ± 1.44 × 10^−2b^	6.35 × 10^−2^ ± 1.12 × 10^−2b^	2.30 × 10^−3^ ± 4.12 × 10^−4b^	2.73 × 10^−1^ ± 2.27 × 10^−2b^	2.74 × 10^−5^ ± 6.49 × 10^−6b^	1.93 × 10^−5^ ± 3.12 × 10^−6b^	N/A
	Hypertensives (*n* = 61)	4.10 × 10^−2^ ± 6.78 × 10^−3ab^	4.31 × 10^−2^ ± 6.27 × 10^−3a^	1.38 × 10^−3^ ± 2.38 × 10^−4a^	8.52 × 10^−2^ ± 1.08 × 10^−2a^	1.83 × 10^−5^ ± 3.05 × 10^−6ab^	1.31 × 10^−5^ ± 1.91 × 10^−6a^	N/A
	Diabetics and hypertensives (*n* = 78)	3.07 × 10^−2^ ± 5.65 × 10^−3a^	4.11 × 10^−2^ ± 5.16 × 10^−3a^	1.18 × 10^−3^ ± 1.82 × 10^−4a^	7.59 × 10^−2^ ± 8.70 × 10^−3a^	1.50 × 10^−5^ ± 2.54 × 10^−6a^	1.25 × 10^−5^ ± 1.57 × 10^−6a^	N/A
	Total (178)	4.19 × 10^−2^ ± 4.64 × 10^−3^	4.66 × 10^−2^ ± 3.99 × 10^−3^	1.49 × 10^−3^ ± 1.47 × 10^−4^	8.99 × 10^−2^ ± 7.33 × 10^−3^	1.88 × 10^−5^ ± 2.09 × 10^−6^	1.42 × 10^−5^ ± 1.21 × 10^−6^	N/A

Gender	Male (*n* = 62)	3.93 × 10^−2^ ± 6.42 × 10^−3a^	4.52 × 10^−2^ ± 6.26 × 10^−3a^	1.43 × 10^−3^ ± 2.20 × 10^-4a^	8.58 × 10^−2^ ± 1.03 × 10^−2a^	1.77 × 10^−5^ ± 2.89 × 10^−6a^	1.37 × 10^−5^ ± 1.90 × 10^−6a^	N/A
	Female (*n* = 116)	4.32 × 10^−2^ ± 6.22 × 10^−3a^	4.73 × 10^−2^ ± 5.10 × 10^−3a^	1.51 × 10^−3^ ± 1.93 × 10^−4a^	9.19 × 10^−2^ ± 9.75 × 10^−3a^	1.94 × 10^−5^ ± 2.80 × 10^−6a^	1.44 × 10^−5^ ± 1.55 × 10^−6a^	N/A
	Total (178)	4.19 × 10^−2^ ± 4.64 × 10^−3^	4.66 × 10^−2^ ± 3.99 × 10^−3^	1.49 × 10^−3^ ± 1.47 × 10^−4^	8.99 × 10^−2^ ± 7.33 × 10^−3^	1.88 × 10^−5^ ± 2.09 × 10^−6^	1.42 × 10^−5^ ± 1.21 × 10^−6^	N/A

Residents	Rural (*n* = 26)	6.57 × 10^−2^ ± 1.46 × 10^−2b^	6.03 × 10^−2^ ± 1.11 × 10^−2a^	2.30 × 10^−3^ ± 5.85 × 10^−4b^	1.28 × 10^−1^ ± 1.99 × 10^−2b^	2.96 × 10^−5^ ± 6.56 × 10^−6b^	1.84 × 10^−5^ ± 3.38 × 10^−6a^	N/A
	Suburban (*n* = 44)	3.02 × 10^−2^ ± 5.21 × 10^−3a^	4.42 × 10^−2^ ± 5.56 × 10^−3a^	1.45 × 10^−3^ ± 2.84 × 10^−4a^	7.59 × 10^−2^ ± 8.98 × 10^−3a^	1.36 × 10^−5^ ± 2.34 × 10^−6a^	1.34 × 10^−5^ ± 1.69 × 10^−6a^	N/A
	Urban (*n* – 108)	4.08 × 10^−2^ ± 6.32 × 10^−3ab^	4.42 × 10^−2^ ± 5.51 × 10^−3a^	1.30 × 10^−3^ ± 1.56 × 10^−4a^	8.62 × 10^−2^ ± 1.03 × 10^−2a^	1.83 × 10^−5^ ± 2.84 × 10^−6ab^	1.34 × 10^−5^ ± 1.67 × 10^−6a^	N/A
	Total (*n* = 178)	4.18 × 10^−2^ ± 4.62 × 10^−3^	4.65 × 10^−2^ ± 3.96 × 10^−3^	1.48 × 10^−3^ ± 1.47 × 10^−4^	8.98 × 10^−2^ ± 7.23 × 10^−3^	1.88 × 10^−5^ ± 2.08 × 10^−6^	1.41 × 10^−5^ ± 1.20 × 10^−6^	N/A

Type of water consumed by participants	Bottled water (*n* = 35)	4.97 × 10^−2^ ± 1.33 × 10^−2a^	5.22 × 10^−2^ ± 1.10 × 10^−2ab^	1.34 × 10^−3^ ± 1.94 × 10^−4a^	1.03 × 10^−1^ ± 2.13 × 10^−2a^	2.23 × 10^−5^ ± 5.96 × 10^−6a^	1.59 × 10^−5^ ± 3.35 × 10^−6ab^	N/A
	Bottled water and for age (*n* = 11)	1.53 × 10^−2^ ± 4.28 × 10^−3a^	3.35 × 10^−2^ ± 1.36 × 10^−2ab^	4.04 × 10^−4^ ± 6.51 × 10^−5a^	4.93 × 10^−2^ ± 1.34 × 10^−2a^	7.00 × 10^−6^ ± 0.92 × 10^−6a^	1.02 × 10^−5^ ± 4.26 × 10^−6ab^	N/A
	Bottled and tap water (*n* = 3)	4.22 × 10^−2^ ± 2.30 × 10^−2a^	1.98 × 10^−2^ ± 7.08 × 10^−3a^	4.36 × 10^−4^ ± 1.33 × 10^−4a^	6.24 × 10^−2^ ± 2.90 × 10^−2a^	1.90 × 10^−5^ ± 1.03 × 10^−6a^	5.98 × 10^−6^ ± 2.15 × 10^−6a^	N/A
	Forage (*n* = 48)	4.18 × 10^−2^ ± 9.17 × 10^−3a^	3.18 × 10^−2^ ± 3.54 × 10^−3ab^	1.274 × 10^−3^ ± 2.11 × 10^−4a^	7.48 × 10^−2^ ± 1.22 × 10^−2a^	1.88 × 10^−5^ ± 4.13 × 10^−6a^	9.63 × 10^−6^ ± 1.08 × 10^−6ab^	N/A
	Stream (*n* = 15)	3.73 × 10^−2^ ± 6.63 × 10^−3a^	7.21 × 10^−2^ ± 1.52 × 10^−2b^	2.063 × 10^−3^ ± 4.65 × 10^−4a^	1.11 × 10^−1^ ± 1.92 × 10^−2a^	1.68 × 10^−5^ ± 2.98 × 10^−6a^	2.19 × 10^−5^ ± 4.62 × 10^−6b^	N/A
	Tap water (*n* = 59)	4.18 × 10^−2^ ± 7.75 × 10^−3a^	5.29 × 10^−2^ ± 7.96 × 10^−3ab^	1.60 × 10^−3^ ± 2.99 × 10^−4a^	9.63 × 10^−2^ ± 1.29 × 10^−2a^	1.88 × 10^−5^ ± 3.49 × 10^−6a^	1.61 × 10^−5^ ± 2.42 × 10^−6ab^	N/A
	Wells (*n* = 5)	5.80 × 10^−2^ ± 4.12 × 10^−2a^	5.28 × 10^−2^ ± 1.76 × 10^−2ab^	4.82 × 10^−3^ ± 2.22 × 10^−3b^	1.16 × 10^−1^ ± 5.28 × 10^−1a^	2.62 × 10^−5^ ± 1.85 × 10^−5a^	1.61 × 10^−5^ ± 5.36 × 10^−6ab^	N/A
	Tot al (*n* = 178)	4.18 × 10^−2^ ± 4.62 × 10^−3^	4.65 × 10^−2^ ± 3.96 × 10^−3^	1.48 × 10^−3^ ± 1.47 × 10^−4^	8.98 × 10^−2^ ± 7.29 × 10^−3^	1.88 × 10^−5^ ± 2.08 × 10^−6^	1.41 × 10^−5^ ± 1.20 × 10^−6^	N/A

*Note:* Values represent mean ± SEM with superscripts a, b representing statistical difference vertically in a column, significant at *p* < 0.05, ANOVA, Duncan multiple comparison post hoc test carried at 95% Confidence interval. Cancer Slope factor (CSF) for heavy metal exposure (oral route), As = 1.5 (mg/kg/day)^−1^, Cd = 0.38 (mg/kg/day)^−1^, Pb: not usually considered carcinogenic (N/A).

Diabetic patients also showed the highest HQ for Cd as compared to hypertensive patients (6.35 × 10^−2^ ± 1.12 × 10^−2^ mg/kg/day vs. 4.31 × 10^−2^ ± 6.27 × 10^−3^ mg/kg/day, *p* < 0.05). Similarly, HQ for lead (Pb) for diabetic patients was significantly higher than that of hypertensive patients (1.38 × 10^−3^ ± 2.38 × 10^−4^ mg/kg/day vs. 2.30 × 10^−3^ ± 4.12 × 10^−4^ mg/kg/day, *p* < 0.05) and comorbid patients (1.18 × 10^−3^ ± 1.82 × 10^−4^ mg/kg/day, *p* < 0.0 5). The HI was the highest in diabetic patients (2.73 × 10^−1^ ± 4.97 × 10^−2^ mg/kg/day, and significantly above that of hypertensive (8.52 × 10^−2^ ± 1.08 × 10^−2^) and comorbid patients (7.59 × 10^−2^± 8.70 × 10^−3^, *p* < 0.05) (Table 7).

Diabetic patients had elevated CR due to As and Cd exposure significantly higher compared to that of hypertensive patients (2.74 × 10^−5^ ± 6.49 × 10^−6^ (mg/kg/day)^−1^ vs. 2.05 × 10^−5^ ± 3.93 × 10^−6^ (mg/kg/day)^−1^, 1.93 × 10^−5^ ± 3.12 × 10^−6^ vs. 1.48 × 10^−5^ ± 2.25 × 10^−6^ (mg/kg/day)^−1^, *p* < 0.05) and was globally consistently above the USEPA acceptable threshold of 1 × 10^−6^ (mg/kg/day)^−1^ (Table 7). No statistically significant difference in HQ for As, Cd and Pb was observed across the different age groups (all *p* > 0.05). Although HI values were below 1, overall HI values were higher in older patients ≥ 85 years, 1.19 × 10^−1^± 1.30 × 10^−2^ (Supporting Table 1).

Rural residents had a higher HQ for As and Pb (6.57 × 10^−2^± 1.46 × 10^−2^ and 2.30 × 10^−3^ ± 5.85 × 10^−4^, *p* < 0.05) compared to patients living in suburban (As 3.02 × 10^−2^ ± 5.21 × 10^−3^ mg/kg/day; Pb 1.45 × 10^−3^ ± 2.84 × 10^−4^ mg/kg/day) and urban (As 4.08 × 10^−2^± 6.32 × 10^−3 ^mg/kg/day; Pb 1.30 × 10^−3^ ± 1.56 × 10^−4 ^mg/kg/day) areas. CR for As and Cd was significantly higher for rural residents (2.96 × 10^−5^± 6.56 × 10^−6^ (mg/kg/day)^−1^) when compared to that of individuals in suburban residents (1.36 × 10^−5^± 2.34 × 10^−6^ (mg/kg/day)^−1^, *p* < 0.005). Overall ≈10^−5^ range was consistently above the USEPA acceptable threshold of 1 × 10^−6^ (mg/kg/day)^−1^ (Table 7).

Participants with streams as their main source of drinking water had significantly higher HQ values for Cd (7.21 × 10^−2^± 1.52 × 10^−2 ^mg/kg/day) as compared to those drinking both tap and bottled water (1.98 × 10^−2^ ± 7.08 × 10^−3^ mg/kg/day, *p* < 0.05), while those who relied on wells had a higher HQ for Pb (4.82 × 10^−3^± 2.22 × 10^−3^ mg/kg/day, *p* < 0.05) as compared to the other groups. HI values remained less than 1 for all groups, while patients who consumed exclusively stream water had a significantly higher CR due to Cd exposure (2.19 × 10^−5^ ± 4.62 × 10^−6^ mg/kg/day)^−1^, *p* < 0.05), when compared to the other groups (Table 7).

#### 3.7.2. CR

The CR from As and Cd exposure exceeded the USEPA threshold of 1 × 10^−6^ (mg/kg/day) ^−1^ across all groups exposing the different groups of patients to a potential long‐term CR (Table 7).

### 3.8. Correlation Analysis

As and Cd exposure strongly correlated with the HI (*r* = 0.815, *p* < 0.001; 0.732, *p* < 0.001, respectively), CR (*r* = 0.955, *p* < 0.001; *r* = 1.000, *p* < 0.001, respectively) and HQ (*r* = 0.954, *p* < 0.001; *r* = 0.960, *p* < 0.001, respectively). Pb exposure correlated with CDI (*r* = 0.976, *p* < 0.001), HQ (*r* = 0.976, *p* < 0.001) and the HI (*r* = 0.578, *p* < 0.001). Diabetes/hypertension correlated negatively with As CR (*r* = −0.158, *p* = 0.035). The HI negatively correlated with GSH (*r* = −0.152, *p* = 0.042) (Table 8).

**TABLE 8:** Pearson correlation analysis between exposure and biomarkers.

Variable	Factor	*R*	*p*
Diabetes/hypertension	Cancer risk of As	−0.158	0.035
	TBARS	0.306	0.001

Residence	CDI Pb	−0.160	0.033
	Pb exposure	−0.148	0.048

Water consumed	CDI Pb	0.174	0.020
	Pb exposure	0.169	0.024

As exposure	Hazard index	0.815	0.001
	Cancer risk of As	0.955	0.001
	Hazard quotient of As	0.954	0.001
	CDI As	0.954	0.001

Cd exposure	Hazard index	0.732	0.001
	Hazard quotient of Cd	0.960	0.001
	Cancer risk of Cd	0.960	0.001

Pb exposure	Hazard index	0.578	0.001

CDI As	Cancer risk of As	1.000	0.001

CDI Cd	Cancer risk of Cd	1.000	0.001

Pb exposure	CDI Pb	0.976	0.001
	Hazard quotient Pb	0.976	0.001
	Hazard index	0.578	0.001

Hazard index	GSH	−0.152	0.042

*Note:* As: arsenic, Cd: cadmium, Pb: lead.

Abbreviations: CDI = chronic daily intake, CR = cancer risk, EDI = estimated daily intake, TC = total cholesterol.

### 3.9. PCT Analysis of Heavy Metal Exposure to HI

**FIGURE 1:**
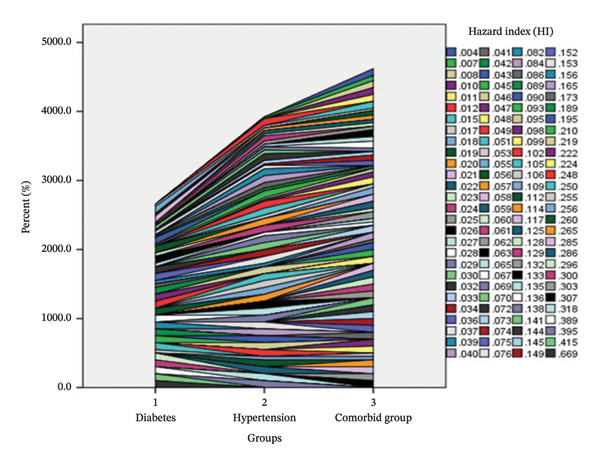
Proportional contribution trend of heavy metals to the hazard index.

Figure 1 illustrates the PCT of As, Cd and Pb to the overall HI across study groups. Cd consistently contributed the largest share of the HI (approximately 55%–65%), followed by As (30%–40%), while Pb contributed minimally (< 10%). The proportional contributions were markedly higher in diabetics, rural residents and participants consuming well or stream water, reflecting the elevated body burden of Cd and As in these groups. By contrast, urban and bottled‐water users showed the lowest proportional contributions (Figure 1).

### 3.10. ROC Analysis and Curves

Significant predictors of worsening diabetic status included CDI As (AUC = 0.604, *p* = 0.047), CDI Pb (AUC = 0.605, *p* = 0.046) and HQ Pb (AUC = 0.606, *p* = 0.044). GSH (AUC = 0.315, *p* < 0.001) and TBARS (AUC = 0.192, *p* < 0.001) were strong predictors of health status and CR among patients (AUC < 0.5) (Table 9, Supporting Figure 4a). For hypertensive patients, TBARS was the only significant predictor (AUC = 0.646, *p* = 0.001) of disease status and health risk, while all other variables showed nonsignificant predictive ability (*p* > 0.05) (Table 9, Supporting Figure 4b). Most heavy metal indices showed poor discrimination for comorbid diabetes‐hypertension health status and for carcinogenic risk (AUC < 0.5, *p* > 0.05) (Table 9, Supporting Figure 4c).

**TABLE 9:** ROC analysis for patients.

Status	Test variable(s)	AUC	p	95% confidence interval	
				Lower bound	Upper bound
Diabetes	Average CDI As	0.604	0.047	0.510	0.698
	Average CDI Pb	0.605	0.046	0.492	0.718
	Hazard quotient of Pb	0.606	0.044	0.493	0.718
	GSH	0.315	0.001	0.221	0.409
	TBARS	0.192	0.001	0.122	0.262

Hypertension	TBARS	0.646	0.001	0.562	0.729

Diabetes and hypertension	Average CDI As	0.379	0.005	0.293	0.464
	Hazard quotient of As	0.380	0.006	0.294	0.465
	As (μg/g creatinine)	0.384	0.008	0.298	0.470

*Note:* The test result variable(s) average CDI As, hazard quotient of As, hazard quotient of Cd, hazard quotient of Pb, hazard index (HI), BMI, As (μg/g creatinine), Pb (μg/g creatinine), GSH, TBARS and PON1, has at least one tie between the positive actual state group and the negative actual state group. Statistics may be biased. ROC analysis performed at 95% CI, significance at *p* < 0.05.

ROC curves for the predictive performance of As, Cd and Pb exposure concentrations, as well as oxidative stress biomarkers (GSH, TBARS, PON1), for distinguishing between diabetic, hypertensive and comorbid patients’ CR, area under the curve (AUC), sensitivity and specificity values are indicated (Supporting Figure 4a, 4b and 4c).

## 4. Discussion

This study assessed urinary levels of arsenic (As), cadmium (Cd) and lead (Pb) among diabetic, hypertensive and comorbid patients and explored their associations with oxidative stress biomarkers and health risk indices. Integrating OLS, PCT and ROC analyses provided mechanistic insights into exposure levels, toxicant burden and biomarker predictivity. The predominance of female participants (65.2%) and middle‐aged adults (55–69 years) aligns with reports that women and older adults are disproportionately affected by NCDs such as diabetes and hypertension in sub‐Saharan Africa [4, 33]. The high representation of farmers and traders further suggests occupational exposure routes, as agricultural and commercial activities often involve contact with contaminated water, fertilizers or consumer products containing trace metals [10]. Moreover, the reliance on nontreated water sources, such as wells and streams, provides plausible environmental exposure pathways for metal ingestion. These patterns collectively reflect socioenvironmental vulnerability among this population, where limited access to safe water and healthcare infrastructures may intensify toxicological risks [34].

OLS regression indicated that water source and residence significantly influenced As and Pb exposure, while other sociodemographic variables showed minimal predictive value. The relatively low *R* ^2^ values (< 0.1) suggest that while environmental and behavioural factors play a role, unmeasured determinants—such as diet, soil contamination and individual metabolic rates—may contribute substantially to exposure variability [15]. The diagnostic plots confirmed model validity, with residuals normally distributed and homoscedastic, supporting the robustness of linear modelling. The consistency of these results highlights that exposure differences are systematic and environmentally driven rather than random, reinforcing the need for localized environmental surveillance programs [6].

### 4.1. Health Implications of Heavy Metal Exposure

Across this study, Cd exposure consistently showed cumulative noncancer and carcinogenic risk. Cd’s dominance is consistent with its long biological half‐life, high bioaccumulation potential and toxicity to the kidney, liver and pancreas. Mechanistic evidence demonstrates that Cd directly obstructs mitochondrial electron transport, increases ROS generation and disrupts lipid catabolism (altered fatty‐acyl metabolism) [35]. The rise in ROS drives the peroxidation of membrane lipids (higher TBARS/MDA) and consumption of thiol antioxidants (GSH), possibly producing the oxidative signature encountered (reduced GSH, altered TBARS) [16]. Because the HI sums the noncancer HQs, Cd’s persistent body burden and relatively unfavourable toxicity parameters make it a large proportional contributor in cumulative risk metrics demonstrated by PCT analyses. In parallel, As exposure, while contributing less proportionally, was strongly correlated with CR indices (*r* = 0.955, *p* < 0.001), and this is consistent with its established genotoxic and carcinogenic properties [36, 37]. Pb, although contributing minimally to PCT, showed significant correlations with CDI, HQ and HI, reflecting its vascular and neurological toxicity [16].

Oxidative imbalance was a central feature in this population, possibly linked to form of therapy, comorbidities and heavy metal exposure. Diabetics exhibited significantly reduced GSH, reflecting consumption of thiols by sustained ROS generation from both hyperglycaemia and heavy metals. TBARS, a measure of lipid peroxidation [38, 39], was significantly elevated in hypertensive patients and predicted hypertensive status with moderate discrimination (AUC = 0.604, *p* < 0.001). This aligns with the role of lipid peroxidation in vascular endothelial dysfunction and hypertension development [26]. PON1 activity, though low, highlights impaired HDL antioxidant defence, which increases susceptibility to atherosclerosis, consistent with reports linking heavy metal exposure to decreased PON1 activity [40]. Inorganic arsenic (As) interferes with insulin signalling, increases insulin resistance through oxidative stress and inflammatory signalling and also displays genotoxic/carcinogenic properties that underline elevated CR for As (> 1 × 10^−6^ mg/kg/day)^−1^ greater than accepted USEPA threshold [15]. Arsenic binds thiols and perturbs redox‐sensitivity signalling (e.g., Nrf2 dysregulation) linking chronic As exposure to higher incidence of cardiometabolic endpoints [41, 42].

Lead perturbs calcium signalling and endothelial function, lowers NO bioavailability via oxidative stress (peroxynitrite formation) and thus promotes hypertension and atherosclerotic processes. Pb’s smaller proportional contribution (< 10% of HI) is consistent with its lower measured concentrations and/or lower contribution to the HI in the different categories of patients [16]. Mechanistically, Pb‐driven oxidative stress can interact synergistically with As and Cd effects to worsen metabolic and vascular pathology [6]. The pathways from exposure to disease involves (i) ingestion/inhalation of As and Cd from contaminated water/food and Pb from environmental sources; (ii) bioaccumulation in target tissues; (iii) disruption of mitochondrial function and thiol homeostasis; (iv) induction of oxidative stress, inflammation and DNA damage; and (iv) progression to β‐cell dysfunction, endothelial damage, insulin resistance and carcinogenesis [16, 35, 36]. The strong correlations between exposure indices and health risk (*p* = 0.01) confirm these pathways.

### 4.2. CR and Non‐CR (HQ and HI)

The calculated EDI and CDI values for all metals were below USEPA reference doses, yet diabetic patients consistently recorded the highest CDI values for As, Cd and Pb. This suggests greater cumulative exposure and potential for long‐term bioaccumulation, which is particularly concerning in individuals with compromised renal function. HQ and HI values remained below 1, indicating no immediate noncarcinogenic risk; however, the higher HI among diabetics (2.73 × 10^−1^ ± 2.27 × 10^−2^) suggests additive toxicity from multiple metals. This finding is consistent with the cumulative risk theory, where combined exposure to multiple contaminants may exert synergistic toxic effects even at subthreshold concentrations [16].

CR estimates for As and Cd exceeded typical USEPA thresholds of 1 × 10^−6^ mg/kg/day^−1^ and PCT analysis identified Cd as the dominant contributor to the group HI (≈55%–65% of HI), with As contributing ∼30%–40% and Pb < 10% contribution to health carcinogenic risk. ROC analysis showed only modest discrimination for individual metal indices in predicting diabetic status (as CDI for As = AUC ≈ 0.60, *p* < 0.05), while TBARs had a fair discrimination for hypertension (AUC 0.646, *p* < 0.05).

### 4.3. Lifetime CR

The calculated CR values for As and Cd exceeded the acceptable threshold of 1 × 10^−6^ (mg/kg/day)^−1^ in all study groups, indicating a measurable excess lifetime CR. In diabetic patients, CR values were 2.74 × 10^−5^ (mg/kg/day)^−1^ for As and 1.93 × 10^−5^ (mg/kg/day)^−1^ for Cd, producing a combined CR of 4.67 × 10^−5^ (mg/kg/day)^−1^. Similarly, hypertensive and comorbid patients had combined CRs of 3.14 × 10^−5^ (mg/kg/day)^−1^ and 2.76 × 10^−5^ (mg/kg/day)^−1^, respectively. While these values are below the upper regulatory benchmark of 1 × 10^−4^ (mg/kg/day)^−1^, they substantially exceed the minimum risk level of 1 × 10^−6^ (mg/kg/day)^−1^, underscoring a non‐negligible carcinogenic burden [31]. Comparable findings of elevated CRs within the 10^−5^–10^−4^ range have been documented in populations exposed to contaminated drinking water [21, 43]. Analysis of proportional contribution demonstrated that Cd accounted for the largest share of cumulative hazard across groups, contributing between 55% and 65%, followed by As (33%–42%) and Pb (< 5%). This distribution highlights Cd and As as the dominant contributors to overall health risks in this population. These results are consistent with prior studies identifying Cd and As as principal drivers of both non‐CR and CR in exposed populations [44, 45]. The associations between exposure indices (CDI, HI and CR) and oxidative stress biomarkers provide biological plausibility for the observed risks. Notably, HI demonstrated a significant negative correlation with reduced GSH, while TBARS and PON1 were altered in relation to exposure indices. These findings align with mechanistic evidence that Cd and As induce oxidative stress by disrupting mitochondrial electron transport and generating ROS, leading to the depletion of antioxidants such as GSH and enhanced lipid peroxidation [46, 47]. Pb, though a minor contributor to cumulative risk, is known to impair calcium signalling and vascular redox balance, which may explain the observed TBARS elevations in certain subgroups [48].

The convergence of elevated heavy metal levels and oxidative stress biomarkers supports a mechanistic model where chronic exposure to Cd, Pb and As amplifies redox imbalance and metabolic dysfunction. Cd and Pb can directly generate ROS and indirectly deplete antioxidant enzymes, while As induces mitochondrial dysfunction and inflammatory cascades [6]. In diabetic and hypertensive patients, pre‐existing oxidative stress and endothelial dysfunction heighten susceptibility to these toxic effects, potentially explaining the observed biomarker trends and elevated HI/CR values [13, 16]. These findings align with the concept of the ‘environmental metabolic syndrome’, where pollutant exposure exacerbates chronic metabolic diseases through oxidative, inflammatory and epigenetic pathways [18].

These findings underscore the need to integrate environmental exposure assessments into NCD management in Cameroon. Cd exposure reduction should be prioritized through improved water safety, regulation of artisanal cookware and community awareness programs. Clinical practices should adopt biomarker panels that combine exposure metrics, oxidative stress markers and clinical parameters to enhance predictive values. Besides clinical and environmental diagnostics, an emphasis should be placed on developing efficient techniques to reduce or remove heavy metal concentrations from food and the environment [2]. Such measures are essential to prevent the detrimental effects of these toxicants on human and aquatic ecosystems. These strategies range from physicochemical systems—including membrane‐based processes, chemical precipitation, electrodialysis, coagulation, flocculation, photocatalytic removal, adsorption and nanomaterial remediation—to bioremediation methods using microbes or plants [2, 49, 50]. Despite their potential, the implementation of these technologies remains limited in Cameroon.

### 4.4. Limitations

This study was a cross‐sectional, limiting causal inference between heavy metal exposure and observed health effects. The sample size, though adequate for statistical comparison, may not fully represent all regional or occupational subgroups. Additionally, urinary normalization using creatinine, while standard, may be influenced by hydration status or renal function variability. Dietary patterns, occupational exposures and environmental concentrations of metals were not directly measured, which could confound exposure assessment. Future studies incorporating longitudinal biomonitoring, environmental sampling and genomic profiling would help clarify temporal and mechanistic relationships.

## 5. Conclusion

This study provides evidence that Cd is the primary driver of cumulative hazard among diabetic and hypertensive patients in Cameroon, with As contributing significantly to CR. Biomarker analyses confirm oxidative stress as a central mechanism linking heavy metal exposure to NCD burden. While PCT highlights priority toxicants for intervention, ROC analysis emphasizes the limitations of single biomarkers in risk prediction. Integrating exposure reduction strategies and biomarker‐informed clinical risk models is recommended to reduce the dual burden of toxicant exposure and NCDs in vulnerable populations. In addition, although non‐CRs (HQ and HI) were below unity, the elevated in diabetics and CRs exceeding 10^−6^ points to a non‐negligible health risk from chronic As and Cd exposure. Public health measures should therefore focus on reducing exposure to these metals, particularly in diabetic populations and rural residents relying on potentially contaminated water sources. Future studies employing longitudinal sampling and physiologically based pharmacokinetic modelling are warranted to strengthen risk estimation in vulnerable populations.

## Author Contributions

Fombat Zenabou Mbebwoh participated in designing and carrying out of the experiments; Muankang Junior Tegha Kum took part in data analysis and drafting of the manuscript; Choumessi Tchewonpi Aphrodite and Kada Sanda Antoine took part in designing and follow‐up of the experiments and revision of the manuscript; Manfo Tsague Faustin Pascal took part in sample analysis and manuscript preparation; Fusi Christian Suh contributed to sample analysis and manuscript revision; and Nantia Akono Edouard contributed to designing and following‐up the study, preparation and revision of the manuscript.

## Funding

This study did not benefit from any funding.

## Conflicts of Interest

The authors declare no conflicts of interest.

## Supporting Information

Additional supporting information can be found online in the Supporting Information section.

## Supporting information


**Supporting Information** Supporting Table 1: Demographic data, heavy metal and oxidative stress levels, chronic daily intake and estimated daily intake assessment and health risk assessment parameters (hazard quotients, hazard index and cancer risk) by age. Supporting Figure 1: Histograms of standardized residuals. Supporting Figure 2: Scatter plots of standardized residuals versus predicted values. Supporting Figure 3: Normal P–P plots of regression standardized residuals. Supporting Figure 4a: ROC curve for diabetes patient’s health risk assessment from heavy metal exposure. ROC curves illustrating the predictive performance of As, Cd and Pb exposure concentrations, as well as oxidative stress biomarkers (GSH, TBARS, PON1), for distinguishing between health status and cancer risk in diabetic patients. Area under the curve (AUC), sensitivity and specificity values are indicated. Supporting Figure 4b: ROC curve for hypertensive patient’s health risk assessment from heavy metal exposure. ROC curves illustrating the predictive performance of As, Cd and Pb exposure concentrations, as well as oxidative stress biomarkers (GSH, TBARS, PON1), for distinguishing between health status and cancer risk in hypertensive patients. Area under the curve (AUC), sensitivity and specificity values are indicated. Supporting Figure 4c: ROC curve for diabetes and hypertensive patient’s health risk assessment from heavy metal exposure. Supporting 10. ROC curves illustrating the predictive performance of As, Cd and Pb exposure concentrations, as well as oxidative stress biomarkers (GSH, TBARS, PON1), for distinguishing between health status and cancer risk in diabetic and hypertensive patients. Area under the curve (AUC), sensitivity and specificity values are indicated.

## Data Availability

The data that support the findings of this study are available from the corresponding author upon reasonable request.
