# Supplementary material for: Exposure to Heavy Metals Arsenic, Cadmium and Lead Exacerbates Carcinogenic and Noncarcinogenic Health Risks Among Diabetic and Hypertensive Patients
Source: J Toxicol. 2026 Jun 30;2026:8564316. doi: 10.1155/jt/8564316 (PMC13318474; doi:10.1155/jt/8564316)
Supplement: Supplementary file 1 — Supporting Information Supporting Table 1: Demographic data, heavy metal and oxidative stress levels, chronic daily intake and estimated daily intake assessment and health risk assessment parameters (hazard quotients, hazard index and cancer risk) by age. Supporting Figure 1: Histograms of standardized residuals. Supporting Figure 2: Scatter plots of standardized residuals versus predicted values. Supporting Figure 3: Normal P–P plots of regression standardized residuals. Supporting Figure 4a: ROC curve for diabetes patient’s health risk assessment from heavy metal exposure. ROC curves illustrating the predictive performance of As, Cd and Pb exposure concentrations, as well as oxidative stress biomarkers (GSH, TBARS, PON1), for distinguishing between health status and cancer risk in diabetic patients. Area under the curve (AUC), sensitivity and specificity values are indicated. Supporting Figure 4b: ROC curve for hypertensive patient’s health risk assessment from heavy metal exposure. ROC curves illustrating the predictive performance of As, Cd and Pb exposure concentrations, as well as oxidative stress biomarkers (GSH, TBARS, PON1), for distinguishing between health status and cancer risk in hypertensive patients. Area under the curve (AUC), sensitivity and specificity values are indicated. Supporting Figure 4c: ROC curve for diabetes and hypertensive patient’s health risk assessment from heavy metal exposure. Supporting 10. ROC curves illustrating the predictive performance of As, Cd and Pb exposure concentrations, as well as oxidative stress biomarkers (GSH, TBARS, PON1), for distinguishing between health status and cancer risk in diabetic and hypertensive patients. Area under the curve (AUC), sensitivity and specificity values are indicated. [file JT-2026-8564316-s001.docx]

**Supplementary Table 1**: Demographic data, heavy metal and oxidative stress levels, chronic daily intake and estimated daily intake assessment and health risk assessment parameters (Hazard Quotients, Hazard Index and Cancer Risk) by age.

| Age groups | | | | | | Male (n, %) | | | | | Female (n, %) | | | Total (n,%) | | | |
| --- | --- | --- | --- | --- | --- | --- | --- | --- | --- | --- | --- | --- | --- | --- | --- | --- | --- |
| 24 – 39 | | | | | | 3 (4.8) | | | | | 6 (5.2) | | | 9 (5.1) | | | |
| 40 – 54 | | | | | | 18 (29.0) | | | | | 34 (29.3) | | | 52 (29.2) | | | |
| 55 – 69 | | | | | | 28 (45.2) | | | | | 52(44.8) | | | 80 (44.9) | | | |
| 70 – 84 | | | | | | 12 (19.4) | | | | | 23(19.8) | | | 35 (19.7) | | | |
| 85 – 104 | | | | | | 1(1.6) | | | | | 1(0.8) | | | 2(1.2) | | | |
| Total | | | | | | 62 (100.0) | | | | | 116 (100.0) | | | 178 (100.0) | | | |
|  | | | | | | | | | | | | | | | | | |
| Age groups (years) | As (µg/g creatinine) | | | Cd (µg/g creatinine) | | | Pb (µg/g creatinine) | | | GSH(mg/dL) | | | TBARS (mg/dL) | | | PON1 (U/L) | |
| 24 – 39  (n = 9) | 1.56± 0.81^a^ | | | 4.40± 2.04^a^ | | | 0.49± 0.21^a^ | | | 54.76±10.09^a^ | | | 2.41 ± 0.35^a^ | | | 0.23 ± 0.07^a^ | |
| 40 – 54  (n = 52) | 0.71± 0.11^a^ | | | 4.46± 0.69^a^ | | | 0.48± 0.08^a^ | | | 49.62±3.39^a^ | | | 2.38 ± 0.17^a^ | | | 0.21 ± 0.02^a^ | |
| 55 – 69  (n = 80) | 1.36± 0.21^a^ | | | 3.93± 0.51^a^ | | | 0.50± 0.08^a^ | | | 50.26±3.40^a^ | | | 2.70 ± 0.14^a^ | | | 0.20 ± 0.02^a^ | |
| 70 – 84 years  (n = 35) | 1.00± 0.21^a^ | | | 4.45± 0.88^a^ | | | 0.38± 0.09^a^ | | | 56.50±6.41^a^ | | | 2.51 ± 0.21^a^ | | | 0.19 ± 0.02^a^ | |
| 85 – 104  (n = 2) | 0.39± 0.23^a^ | | | 8.94± 3.11^a^ | | | 0.52± 0.32^a^ | | | 54.90±6.90^a^ | | | 2.72 ± 0.27^a^ | | | 0.34 ± 0.09^a^ | |
| Tot al  (n = 178) | 1.10± 0.12 | | | 4.27± 0.36 | | | 0.47± 0.05 | | | 51.58±2.26 | | | 2.56 ± 0.92 | | | 0.20 ± 0.01 | |
|  | | | | | | | | | | | | | | | | | |
|  | Chronic daily intake (CDI) (mg/kg/day) | | | | | | | | | Estimated daily intake (mg/kg/day) | | | | | | | |
| Age groups | As (mg/kg/day) | | Cd (mg/kg/day) | | | | Pb (mg/kg/day) | | | As (mg/kg/day) | | | Cd (mg/kg/day) | | | Pb (mg/kg/day) | |
| 24 – 39 years  (n = 9) | 1.68 × 10^-5^ ± 6.93 × 10^-6 a^ | | 3.76 × 10^-5^ ± 1.36 × 10^-5^ | | | | 5.56 × 10^-6^ ± 2.05 × 10^-6^ | | | 1.68 × 10^-5^ ± 6.99 × 10^-6 a^ | | | 4.70 × 10^-5^ ± 1.70 × 10^-5 a^w | | | 5.56 × 10^-6^ ± 2.05 × 10^-6 a^ | |
| 40 – 54 years  (n = 52) | 8.12 × 10^-6^ ± 1.37 × 10^-6 a^ | | 3.887 × 10^-5^ ± 6.06 × 10^-6 a^ | | | | 5.28 × 10^-6^ ± 9.33 × 10^-7 a^ | | | 8.14 × 10^-6^ ± 1.37 × 10^-6 a^ | | | 4.84 × 10^-5^ ± 7.58 × 10^-6 a^ | | | 5.28 × 10^-6^ ± 9.33 × 10^-7 a^ | |
| 55 – 69 years  (n = 80) | 1.51 × 10^-5^ ± 2.38 × 10^-6 a^ | | 3.36 × 10^-5^ ± 4.13 × 10^-6 a^ | | | | 5.35 × 10^-6^ ± 7.97 × 10^-7 a^ | | | 1.51 × 10^-5^ ± 2.38 × 10^-6 a^ | | | 4.20 × 10^-5^ ± 5.16 × 10^-6 a^ | | | 5.36 × 10^-6^ ± 7.97 × 10^-7 a^ | |
| 70 – 84 years  (n = 35) | 1.26 × 10^-5^ ± 3.48 × 10^-6 a^ | | 4.07 × 10^-5^ ± 8.79 × 10^-6 a^ | | | | 4.50 × 10^-6^ ± 1.16 × 10^-6 a^ | | | 1.26 × 10^-5^ ± 3.47 × 10^-6 a^ | | | 5.09 × 10^-5^ ± 1.20 × 10^-5 a^ | | | 4.50 × 10^-6^ ± 1.16 × 10^-6 a^ | |
| 85 – 104 years  (n = 2) | 5.00 × 10^-6^ ± 3.00 × 10^-6 a^ | | 8.07 × 10^-5^ ± 1.97 × 10^-5 a^ | | | | 6.55 × 10^-6^ ± 4.44 × 10^-6 a^ | | | 4.87 × 10^-6^ ± 3.20 × 10^-6 a^ | | | 1.01 × 10^-4^ ± 2.46 × 10^-5 a^ | | | 6.55 × 10^-6^ ± 4.44 × 10^-6 a^ | |
| Tot al  (n = 178) | 1.25 × 10^-5^ ± 1.39 × 10^-6^ | | 3.72 × 10^-5^ ± 3.17 × 10^-6^ | | | | 5.19 × 10^-6^ ± 5.13 × 10^-7^ | | | 1.25 × 10^-5^ ± 1.38 × 10^-6^ | | | 4.65 × 10^-5^ ± 3.96 × 10^-6^ | | | 5.19 × 10^-6^ ± 5.13 × 10^-7^ | |
|  | | | | | | | | | | | | | | | | | |
|  | | Health Hazard quotient (HQ) | | | | | | | Hazard Index (HI) | | | Cancer Risk (CR) | | | | | |
| Age groups | | As | | | Cd | | | Pb | Sum (HQ) (HI) | | | As ((mg/kg/day)⁻¹) | | | Cd ((mg/kg/day)⁻¹) | | Pb((mg/kg/day)⁻¹) |
| 24 – 39 years(n = 9) | | 5.61 × 10^-2^ ± 2.33 × 10^-2 a^ | | | 4.71 × 10^-2^ ± 1.70 × 10^-2 a^ | | | 1.59 × 10^-3^ ± 5.87 × 10^-4 a^ | 1.05 × 10^-1^ ± 4.04 × 10^-2 a^ | | | 2.53 × 10^-5^ ± 1.05 × 10^-5 a^ | | | 1.43 × 10^-5^ ± 5.16 × 10^-6 a^ | | N/A |
| 40 – 54 years (n = 52) | | 2.71 × 10^-2^ ± 4.57 × 10^-3 a^ | | | 4.84 × 10^-2^ ± 7.58 × 10^-3 a^ | | | 1.51 × 10^-3^ ± 2.67 × 10^-4 a^ | 7.70 × 10^-2^ ± 9.60 × 10^-3 a^ | | | 1.22 × 10^-5^ ± 2.06 × 10^-6 a^ | | | 1.47 × 10^-5^ ± 2.30 × 10^-6 a^ | | N/A |
| 55 – 69 years  (n = 80) | | 5.03 × 10^-2^ ± 7.93 × 10^-3 a^ | | | 4.20 × 10^-2^ ± 5.16 × 10^-3 a^ | | | 1.53 × 10^-3^ ± 2.28 × 10^-4 a^ | 9.39 × 10^-2^ ± 1.10 × 10^-2 a^ | | | 2.26 × 10^-5^ ± 3.57 × 10^-6 a^ | | | 1.27 × 10^-5^ ± 1.57 × 10^-6 a^ | | N/A |
| 70 – 84 years  (n = 35) | | 4.19 × 10^-2^ ± 1.16 × 10^-2 a^ | | | 5.09 × 10^-2^ ± 1.10 × 10^-2 a^ | | | 1.27 × 10^-3^ ± 3.31 × 10^-4 a^ | 9.39 × 10^-2^ ± 2.11 × 10^-2 a^ | | | 1.88 × 10^-5^ ± 5.21 × 10^-6 a^ | | | 1.56 × 10^-5^ ± 3.34 × 10^-6 a^ | | N/A |
| 85 – 104 years  (n = 2) | | 1.68 × 10^-2^ ± 1.05 × 10^-2 a^ | | | 1.01 × 10^-1^ ± 2.50 × 10^-2 a^ | | | 1.87 × 10^-3^ ± 1.27 × 10^-3 a^ | 1.19 × 10^-1^ ± 1.30 × 10^-2 a^ | | | 7.32 × 10^-6^ ± 4.79 × 10^-6 a^ | | | 3.07 × 10^-5^ ± 7.49 × 10^-6 a^ | | N/A |
| Tot al  (n = 178) | | 4.18 × 10^-2^ ± 4.12 × 10^-3^ | | | 4.65 × 10^-2^ ± 4.00 × 10^-3^ | | | 1.48 × 10^-3^ ± 1.47 × 10^-4^ | 8.98 × 10^-2^ ± 7.29 × 10^-3^ | | | 1.88 × 10^-5^ ± 2.08 × 10^-6^ | | | 1.41 × 10^-5^ ± 1.20 × 10^-6^ | | N/A |

Values on the table represent frequency distribution of demographic of respondents represented as percentages, with mean ± SEM with superscripts a, b representing statistical difference vertically in a column, significant at p < 0.05, ANOVA, Duncan multiple comparison post hoc test carried at 95% Confidence interval. Cancer Slope factor (CSF) for heavy metal exposure (oral route), As = 1.5 (mg/kg/day) ⁻¹,Cd = 0.38 (mg/kg/day) ⁻¹, Pb: not usually considered carcinogenic (N/A).

**
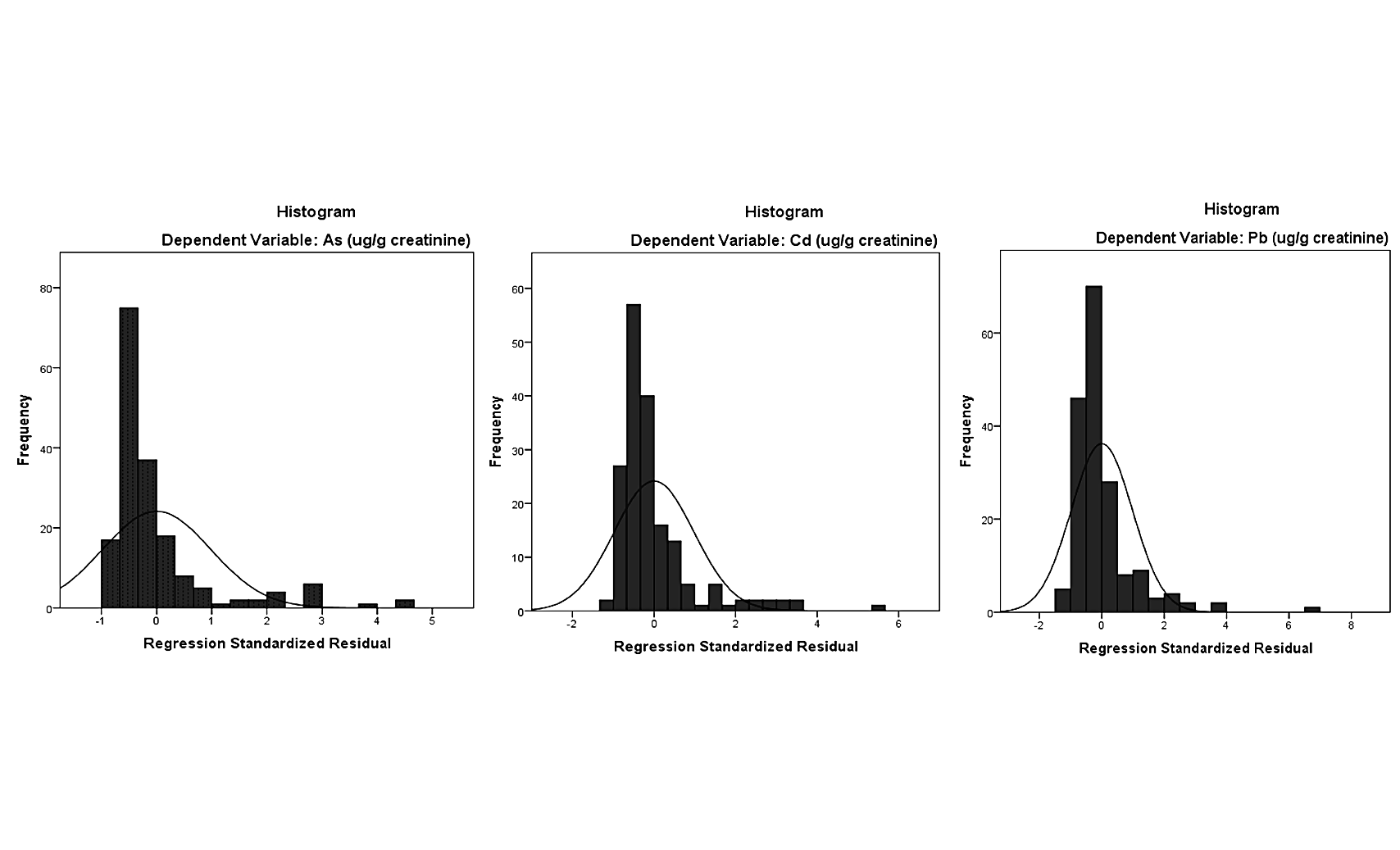
**

**Supplementary Figure 1:** Histograms of standardized residuals.

***
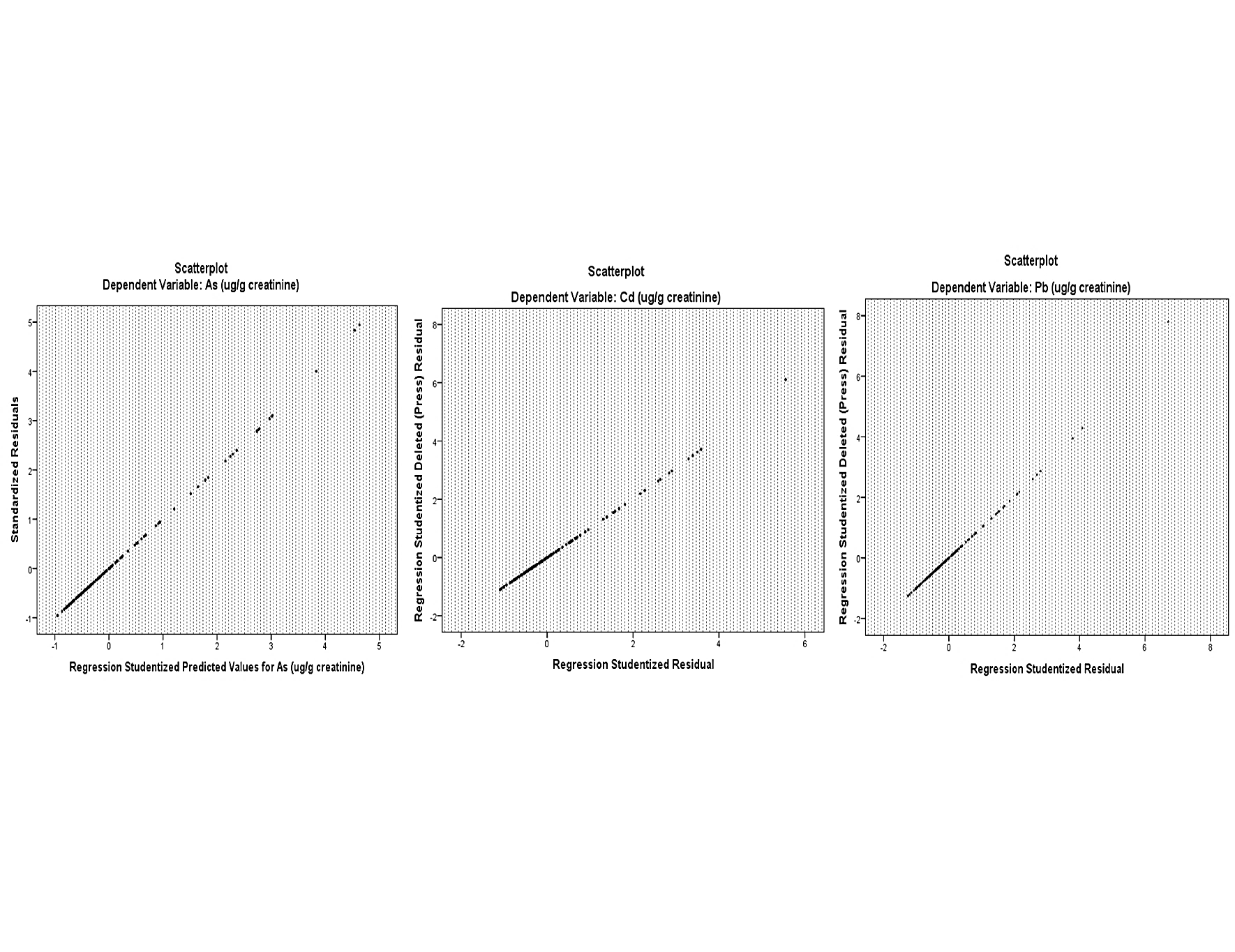
*****Supplementary Figure 2:** Scatterplots of standardized residuals versus predicted values

***
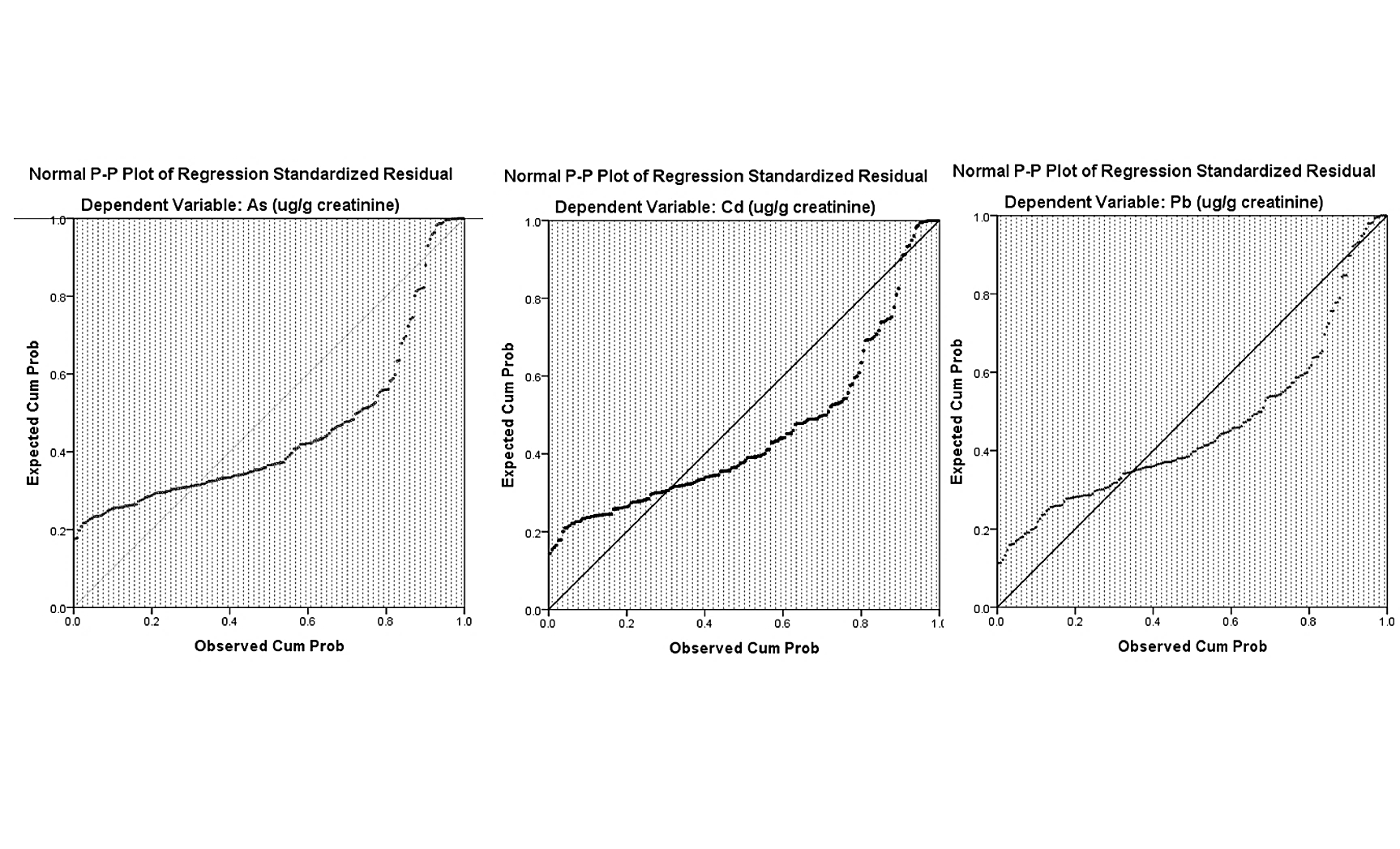
***

**Supplementary Figure 3:** Normal P–P plots of regression standardized residuals


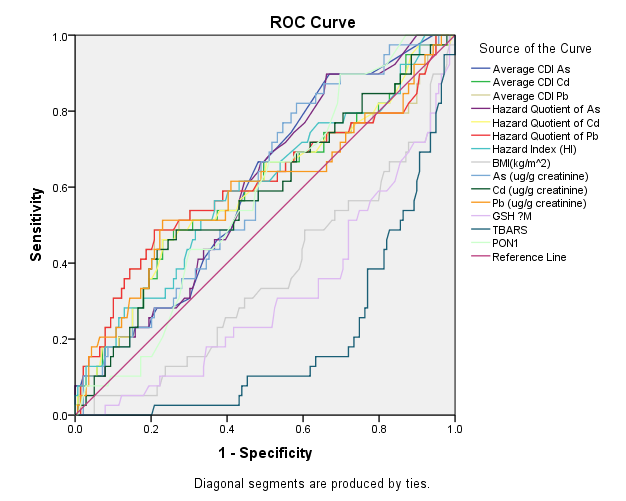
**Supplementary Figure 4a**: ROC curve for diabetes patient’s health risk assessment from heavy metal exposure.


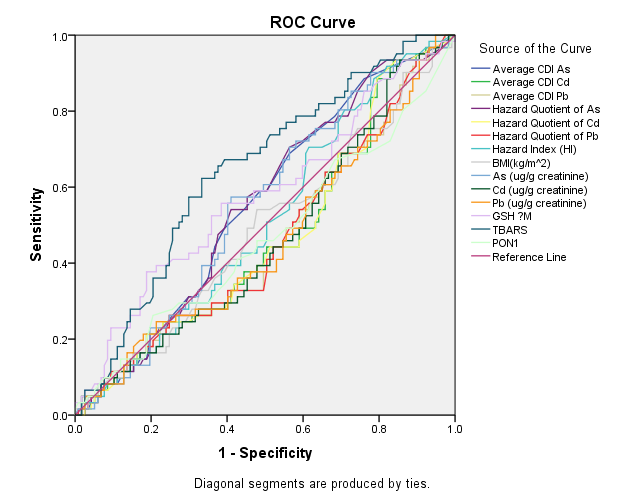
**Supplementary Figure 4b**: ROC curve for hypertensive patient’s health risk assessment from heavy metal exposure.


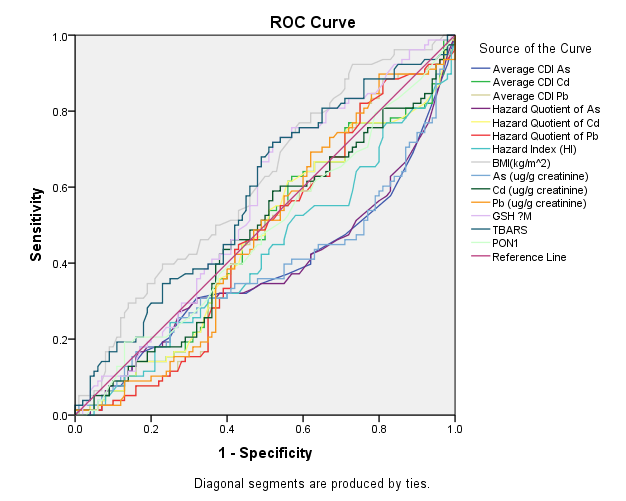


**Supplementary Figure 4c:** ROC curve for diabetes and hypertensive patient’s health risk assessment from heavy metal exposure.
